# Evolution of reproductive mode variation and host associations in a sexual-asexual complex of aphid parasitoids

**DOI:** 10.1186/1471-2148-11-348

**Published:** 2011-12-01

**Authors:** Christoph Sandrock, Bettina E Schirrmeister, Christoph Vorburger

**Affiliations:** 1 Institute of Evolutionary Biology and Environmental Studies, University of Zürich, Winterthurerstrasse 190, 8057 Zürich, Switzerland; 2 Swiss Bee Research Centre, Agroscope Liebefeld-Posieux ALP, Schwarzenburgstrasse 161, 3003 Bern, Switzerland; 3 Institute of Integrative Biology, ETH Zürich, Switzerland, and EAWAG, Swiss Federal Institute of Aquatic Science and Technology, Überlandstrasse 133, 8600 Dübendorf, Switzerland

## Abstract

**Background:**

The *Lysiphlebus fabarum *group is a taxonomically poorly resolved complex of aphid parasitoids, presently split into three described species that comprise sexual (arrhenotokous) and asexual (thelytokous) lineages of unknown relationship. Specifically, it is unclear how asexuals evolved from sexuals in this system, to what extent reproductive modes are still connected by genetic exchange, how much the complex is structured by geography or by host-associated differentiation, and whether species designations are valid. Using a combination of population genetic and phylogenetic approaches, we addressed these issues in a comprehensive sample of parasitoid wasps from across Europe.

**Results:**

Asexual reproduction predominated in parasitoids of the *L. fabarum *group, with asexual populations exhibiting high genotypic diversity. Sexual populations were only common in southern France; elsewhere sexual reproduction was restricted to specific aphid hosts. Although reproductive modes were aggregated on the mitochondrial genealogy and significantly differentiated at nuclear microsatellite loci, there was clear evidence for genetic exchange, especially on hosts attacked by sexual and asexual parasitoids. The microsatellite data further revealed that parasitoids collected from certain host aphids were significantly differentiated, yet the mitochondrial sequence variation across the entire *L. fabarum *group did not exceed 1.32% and exhibited a very shallow topology. Morphological characters used for delineation of described species were found to be phylogenetically non-conservative.

**Conclusions:**

Our results suggest that the sexual-asexual *L. fabarum *group represents a young complex of lineages with incomplete isolation between reproductive modes. We propose three mechanisms of genetic exchange that may jointly explain the high genotypic diversity observed in asexual parasitoids: (*i*) the formation of new asexual lineages via 'contagious parthenogenesis', (*ii*) introgression from sexual lineages through matings between sexual males and thelytokous females, and (*iii*) 'cryptic sex' within asexuals, mediated by rare males that thelytokous lines are known to produce spontaneously. The partially strong differentiation among wasps collected from different aphids suggests that host specialization can evolve readily in these parasitoids. Finally, we conclude that in the light of our data, the current taxonomic division of the *L. fabarum *group into three species cannot be upheld.

## Background

Apart from a few notable exceptions [1-3], asexual organisms tend to be young on an evolutionary time scale. This supports the general assumption that genetic exchange through sex and recombination is required for long-term persistence [e.g. [4]. Because of their recent origin, most asexual organisms have close relatives that are sexual. Such sexual-asexual complexes represent promising models to study the relative costs and benefits of sexual vs. asexual reproduction in an ecological context. An important aspect for these comparisons is the genetic variation present in sexual and asexual populations. Genotypic diversity in asexuals can be surprisingly high if transitions to asexuality occur frequently or if asexual lineages acquire new variation through some form of 'cryptic sex' [e.g. [5,6]].

Parasitoids of the genus *Lysiphlebus *(Hymenoptera: Braconidae: Aphidiinae) represent an interesting system to address these issues. Like other Hymenopterans, they typically reproduce by arrhenotoky [7]; unfertilized eggs develop into haploid males and fertilized eggs develop into diploid females. However, all-female lineages are common in a poorly resolved group of *Lysiphlebus*-taxa from the Palearctic that mainly attacks aphids of the genera *Aphis *and *Brachycaudus *[8,9]. They reproduce by thelytoky, the production of diploid females without fertilization [10]. This group comprises three morphologically described species: *L. fabarum *(Marshall 1896), *L. cardui *(Marshall 1896) and *L. confusus *(Tremblay & Eady 1978). Their separating morphological characters are summarized in Table 1. Since the first genetic study of this group found little support for this distinction [8], we will call the whole complex the *L. fabarum *group (LFG) and refer to the three taxa as morphotypes *Lfa*, *Lca *and *Lco*. All three morphotypes are reported to contain sexual and asexual populations [8].

**Table 1:** Morphological key to distinguish traditionally recognized species within the *Lysiphlebus fabarum *group.

		Setae				
		
		Hind-femora			Apical margins of forewing	
			
Species	Abbreviation	Adpressed		Erect	Short	Long
*L. fabarum*	*Lfa*	**×**			**×**	
*L. confusus*	*Lco*	**×**	or	**×**		**×**
*L. cardui*	*Lca*			**×**	**×**	

Summarized and modified according to [22,24,102,106-109].

Based on a close association between reproductive mode and mitochondrial DNA variation, Belshaw *et al*. [8] concluded that only few transitions to thelytokous reproduction took place in *Lysiphlebus*. However, a nuclear DNA marker showed no association with reproductive mode or the mitochondrial genealogy. This suggested that rare or cryptic sex occasionally occurs in thelytokous *Lysiphlebus*, and that thelytoky may be under nonnuclear control in these wasps [8]. This could be the case if thelytoky in *Lysiphlebus *was induced by *Wolbachia *or other microbial symbionts [11-13], but *Wolbachia *does not seem to occur in *Lysiphlebus *([8,14] & own unpubl. data). That microbes are not involved is also supported by the cytological mechanism of diploidy restoration, which Belshaw & Quicke [15] identified as equivalent to central fusion automixis [see also [16]]. This mechanism is inconsistent with any currently known form of microbe-induced parthenogenesis in insects [11-13]. Indeed, it was shown recently that counter to original conjectures, thelytoky in *L. fabarum *is under nuclear control [17]. Crossing experiments using haploid males that are produced occasionally by thelytokous lines revealed that a single, recessive allele determines thelytoky, similar to observations made in the Cape honeybee [18]. This suggests one way by which asexual populations can acquire genetic variation: thelytoky-inducing alleles may be spread by rare males from thelytokous lineages and convert sexual into asexual lineages. This process termed 'contagious parthenogenesis' [5] is also observed e.g. in *Daphnia *[19,20], rotifers [21] or aphids [6]. However, contagious parthenogenesis would recruit new mitochondrial variation from sexual into asexual populations and is thus difficult to reconcile with the association between mitochondrial haplotypes and reproductive mode reported by Belshaw *et al*. [8]. Therefore, one aim of the present study was to carefully study the genetic relationships among populations with different reproductive modes and try to infer the amount and possible routes of genetic exchange between them.

The potential for genetic exchange in the *Lysiphlebus *system is also influenced by the degree of host specialization that different lineages exhibit. Numerous host species are exploited by the LFG [22,23]. Field surveys suggest that different morphotypes tend to differ in their host associations and that sexual lineages are disproportionately common on certain hosts [8,23,24]. These observations suggest a high degree of host specialization in the LFG. This would be in line with findings on other parasitoids of herbivorous insects, showing that the strong specialization of herbivores cascades upward to the trophic level of parasitoids [25-30]. On the other hand, the few population genetic studies on aphidiine parasitoids available so far suggest little host-associated differentiation (HAD) [31-34]. This might be different for *Lysiphlebus *wasps, however, because most taxa use chemical camouflage to be able to attack ant-defended colonies of aphids [35,36], which might exert stronger selection for specialization. Another aim of this study was thus to assess the degree of host specialization exhibited by sexual and asexual *Lysiphlebus *parasitoids.

We used highly polymorphic nuclear markers (microsatellites) and mitochondrial DNA sequences of two protein coding genes to assess phylogenetic relationships and the genetic population structure of a comprehensive sample of parasitoids from the LFG. Several complementary analytic approaches were applied to these data to address the following questions: (*i*) How is genetic variation partitioned among sexual and asexual lineages in the LFG? (*ii*) Which conclusions can be drawn about the evolutionary history of reproductive modes and putative mechanisms of cryptic sex? (*iii*) Is there any evidence for HAD within the LFG? (*iv*) Does the genetic structure exhibit geographic patterns? (*v*) Is there genetic support for the present taxon definitions according to morphological traits? (*vi*) Do the combined answers to these questions have implications for the taxonomy of the LFG?

## Methods

### Field sampling and specimen characterization

Parasitoids were sampled at 15 locations in 6 countries across Europe (Table 2). We harvested visibly parasitized ('mummified') host colonies of the following aphid-plant complexes, known to be foraged by the *L. fabarum *group (LFG) [22,23]: *Aphis fabae fabae *(*Vicia faba*, *Chenopodium album*, *Beta sp*.), *A. f. cirsiiacanthoidis *(*Cirsium arvense*), *A. hederae *(*Hedera helix*)*, A. urticata *(*Urtica dioica*)*, A. ruborum *(*Rubus sp*.), *A. farinosa *(*Salix sp*.) and *Brachycaudus cardui *(*Carduus sp*.). Several unspecified hosts of the genus *Aphis *were sampled occasionally from various plants (*Rumex sp*., *Galium sp*., *Tanacetum vulgare*, *Matricaria chamomilla*, *Solanum sp*., *Viburnum opulus, Lactuca sp*., *Nerium oleander*) and are combined within the group *Aphis sp*. To minimize biases due to thelytokous propagation we only collected host colonies from different plants separated by at least 10 meters. Host colonies were stored in aerated containers and brought to the laboratory. Hatched LFG parasitoids were sexed, counted and morphologically categorized as detailed in Table 1.

**Table 2:** Sampling summary of European *Lysiphlebus fabarum *group parasitoids.

					Host species								Morphotype			Mode			
								
	Geographic area	Area label	Coordinates	Total	*Aff*	*Afc*	*Aur*	*Ahe*	*Aru*	*Afa*	*Asp*	*Bca*	*Lfa*	*Lco*	*Lca*	Sex	Asex	Asex MLG	*R*
	Toscana (I)	*A*	44°06' N, 9°55'E	14	7	-	-	1	-	-	6	-	11	2	1	-	14	3	0.154
	Emilia-Romagna (I)	*B*	44°07'N, 12°15'E	1	1	-	-	-	-	-	-	-	-	1	-	-	1	1	0.000
	Camargue (F)	*C*	43°40'N, 4°08'E	43	3	-	-	21	14	-	-	5	31	12	-	28	15	9	0.857
	Côte d'Azur (F)	*D*	43°16'N, 6°31'E	54	13	1	-	18	16	-	6	-	22	30	2	18	36	11	0.528
	Valais (CH)	*E*	46°08'N, 7°06'E	42	2	7	3	15	15	-	-	-	24	18	-	16	26	5	0.488
	Ticino (CH)	*F*	46°08'N, 8°56'E	51	13	-	4	7	23	1	3	-	14	21	16	-	51	10	0.180
	Brittany (F)	*G*	48°07'N, 1°45'E	38	4	6	2	9	10	1	6	-	19	10	9	-	38	21	0.541
	Grisons (CH)	*H*	46°52'N, 9°32'E	39	3	6	4	4	16	2	4		18	16	5	3	36	13	0.486
	Bohemia (CZ)	*I*	48°54'N, 14°29'E	66	20	13	7	11	-	8	7	-	33	10	23	-	66	20	0.292
	Basel-area (CH)	*J*	47°29'N, 7°37'E	60	6	8	7	23	11	1	4	-	37	9	14	11	49	21	0.525
	Hesse (D)	*K*	50°10'N, 9°09'E	72	4	26	4	17	15	-	2	3	50	10	12	19	53	17	0.486
	Dithmarschen (D)	*L*	53°55'N, 9°09'E	137	27	36	1	43	20	4	6	-	79	5	53	-	137	38	0.272
	St. Margrethen (CH)	*M*	47°27'N, 9°38'E	24	5	2	-	7	10	-	-	-	15	7	2	-	24	9	0.333
	Cambridge (UK)	*N*	52°13'N, 0°02'E	59	25	7	-	7	13	3	1	3	39	7	13	3	56	17	0.328
	Zurich-area (CH)	O	47°22'N, 8°29'E	211	3	14	4	153	25	7	2	3	175	23	13	107	104	37	0.689
*Lfa*				567	82	39	20	317	78	-	17	14				202	368	103	0.537
*Lco*				181	13	9	5	12	111	27	4	-				3	175	52	0.305
*Lca*				163	41	78	12	7	5	-	20	-				-	163	25	0.148
Sex				205	1	3	-	180	5	-	2	14							1.000
Asex				706	135	123	37	156	189	27	38	-							0.254
Asex MLG				180	51	43	20	50	56	20	26	-							
Total				911	136	126	37	336	194	27	41	14	567	181	163	205	706	180	
*R*				0.542	0.378	0.360	0.528	0.687	0.311	0.731	0.675	1							

Overview of geographic locations, corresponding labels and approximate sampling coordinates. Total sample sizes (total) and numbers of samples specified for host species (see below), morphotypes (see Table 1), reproductive mode (Sex, arrhenotokous; Asex, thelytokous) and unique asexual multilocus genotypes (Asex MLG) are presented per region and in total. Sampled hosts were the following: *Aphis fabae fabae *(*Aff*), *A. f. cirsiacanthoidis *(*Afc*), *A. urticata *(*Aur*), *A. hederae *(*Ahe*), *A. ruborum *(*Aru*), *A. farinosa *(*Afa*), unspecified *Aphis *(*Asp*) and *Brachycaudus cardui *(*Bca*). Microsatellite genotypic diversity is estimated as *R *and shown for *Lysiphlebus *parasitoids corresponding to individual sites, host species, morphotypes and reproductive modes, respectively.

### Determination of reproductive modes

Reproductive modes of LFG samples were determined using a combination of the following approaches. First, sex ratios were recorded. In general, the absence of males suggests asexual reproduction for samples containing large numbers of individuals. Yet, asexual lineages were shown to occasionally produce males [17]. Second, up to three virgin females were obtained by isolating single aphid mummies. They were individually allowed to parasitize nymphs of *A. f. fabae *cultured on broad beans (*V. faba *var. 'Scirocco') to infer their reproductive mode, and then genotyped. Thelytokous virgins produce only daughters, arrhenotokous virgins only sons. For samples in which both sexes had emerged before reaching the laboratory, virgins were isolated from cultured F_1 _offspring. When all LFG parasitoids either died during transport or failed to reproduce under laboratory conditions, several females and (if present) males were genotyped for those samples. Unique microsatellite multilocus genotypes (MLGs) of each individual indicate sexual reproduction whereas multiple females sharing identical MLGs were considered as asexuals.

### General statistical analyses

Sex ratio data from field samples were analysed in R 2.8.1 [37] with a generalized linear model and a quasibinomial error distribution to account for overdispersion [38]. We tested for the effects of location, host and the location × host interaction as fixed effects. Location was treated as a fixed effect because we analysed the data under the *a priori *expectation that LFG parasitoids differ geographically in reproductive mode [see [8]]. We excluded the group of unspecified hosts for this analysis. Further, we tested whether different morphotypes were associated with distinct host aphids by performing a Fisher's exact test based on cross table comparisons using SPSS v17.0. Similarly, we tested for significant differences in the occurrence of morphotypes among locations.

### Microsatellite genotyping and basic analyses

Preparations of genomic DNA were performed as detailed in Sandrock *et al*. [39] and then stored at -20°C until use. Microsatellite genotyping was conducted using a published multiplex protocol [39]. Marker Lysi01 was discarded from multiplex set 1 [39] due to inconsistent and ambiguous allele scores and multiplex set 2 was supplemented by marker Lysi5a12 developed for *L. testaceipes *[40]. We determined fragment sizes on an ABI 3730 sequencer and allele scoring was performed using the GeneMapper software v3.7.

We calculated allelic and genotypic diversity using *GenClone *[41] and assessed the probabilities (*p*_sex_) of repeatedly detected MLGs to be produced by independent sexual events [42]. The frequency distribution of the pairwise number of allele differences of all MLGs was plotted as a genetic distance metric to address the possibility that not all unique genotypes stem from sexual events but also from allele scoring errors or somatic mutations within asexual lineages [41,42]. This frequency distribution was bimodal with the smaller peak representing a number of only slightly distinct genotypes. Examination of these pairs of MLGs suggested that this effect was caused by two hypervariable loci displaying extraordinarily high allelic variation, i.e., Lysi02 and Lysi10 (97 and 64 alleles, respectively). Re-estimating *p*_sex _without these loci clearly suggested that all corresponding pairs of MLGs were members of the same asexual lineage and not derived from distinct reproductive events. Both highly variable markers were therefore discarded from all further analyses. Their exclusion resulted in a unimodal frequency distribution of genetic distances.

We calculated genotypic diversity per sample location using the diversity index *R *[43] as implemented in *GenClone*. We checked for random mating at individual loci within sexual populations and estimated marker linkage in Arlequin v3.11 [44]. Allelic diversity, observed heterozygosities and *F*-statistics for single loci were assessed in FSTAT [45]. *A priori*, we expected differences for different morphotypes (putatively distinct species) and reproductive modes, because central fusion automixis in asexuals might result in increased homozygosity [46]. Thus, we analysed different morphotypes within reproductive modes separately according to geographic origin. Analyses were performed with single copy MLGs per group (e.g. host origin) to avoid violations of basic population genetic assumptions (for rationale see [47,48]). Significance levels were adjusted using the sequential Bonferroni correction [49].

### Taxon sampling and DNA sequencing

We subjected 37 arrhenotokous and 118 distinct thelytokous microsatellite MLGs of the LFG to mitochondrial sequencing to reconstruct phylogenetic relationships. This selection covered the total range of morphotypes from each host species and each geographic origin. In addition, for each of 15 thelytokous MLGs in this selection we sequenced a second individual that possessed the same MLG but was collected from a different host species and a different location. As outgroups we included specimens of two purely sexual species of the same genus, the Palaearctic *L. hirticornis *and the Nearctic *L. testaceipes*. Further, one specimen each of *Adialytus salicaphis*, *Diaeretiella rapae *and *Aphidius colemani *was included.

We sequenced two mitochondrial genes partially. We used the primer pair LCO 1490 and HCO 2198 [50], to amplify a 658 bp fragment of the cytochrome *c *oxidase subunit I (COI, 'barcode region'). In addition, we amplified the almost total length sequence (644 bp) of the adenosine triphosphate synthase subunit 6 gene (ATP6) using the new primers AATP6F (5'-TTTTCWATTTTTGATCCWTCWAC-3') and AATP6CO3R (5'- CTTACTAAATGATAAGGATG-3'). PCR reactions were performed in a Techne TC-512 thermocycler in 9 μL volumes containing 1× QIAGEN Multiplex PCR MasterMix, including PCR buffer (3 mM MgCl_2_), a dNTP mix and HotStar Taq DNA polymerase, 2.3 μL of genomic DNA and 10 μM of each primer. COI amplifications were conducted using cycling conditions described in Hebert *et al*. [51] complemented by an initial denaturation of 15 min at 95°C and an extended final cycle at 72°C (30 min). Thermal regime for ATP6 amplifications consisted of an initial denaturation of 15 min at 95°C and a round of 40 cycles each composed of 40 sec at 94°C, 1 min at 50°C and 1 min at 72°C, finally completed by a cycle of 30 min at 72°C. PCR products were checked on 2% agarose gels using GelRed™ staining and purified using the *ExoSAP-IT^® ^*kit. Both strands of each fragment were cycle sequenced using ABI BigDye Terminator reagents and analyzed on an ABI 3730 sequencer.

### Sequence analyses and phylogenetics

Sequences were manually edited in Chromas Lite v 2.01 (Technelysium Pty. Ltd., 1998-2005) and aligned using Clustal W [52] as implemented in BioEdit v7.0.9.0 [53]. All sequences were indel-free. We used MEGA v4.0.2 [54] to evaluate variable sites and to confirm continuous open reading frames in both protein-coding genes to exclude nuclear pseudogenes [55,56]. Numbers of haplotypes were computed in DNASP v5.10 [57].

Preliminary tree reconstruction of COI and ATP6 produced very similar results. Therefore, sequences of both genes were concatenated to maximize phylogenetic signals (1302 characters). Phylogenetic reconstruction was carried out using maximum parsimony (MP), computed with PAUP* 4.0b10 [58], maximum likelihood (ML), performed with GARLI v0.96 [59], and Bayesian inference (BI), conducted in MrBayes 3.1 [60]. Heuristic parsimony searches were performed for 500 replicates using random stepwise addition, TBR branch swapping and collapse of zero length branches. Results were summarized using a strict consensus and robustness was estimated with bootstrap analyses. For ML and BI we identified GTR + G + I [61,62] as the best-fitting model of sequence evolution based on the Akaike Information Criterion as implemented in Modeltest 3.5 [63], with parameters estimated by the program. Fifty replicate heuristic searches were performed for ML analysis resulting in very similar log-likelihoods. Bootstrap values were obtained from 2*500 re-samplings of the data set. BI was conducted running two MC^3 ^searches, each with one cold and three heated chains. Starting with a random tree, analyses were run for 20 million generations each, with trees being sampled every 100 generations. Convergence of the parameters was confirmed with the programs AWTY [64] and Tracer v1.4.1 [65]. The first 4000 trees were discarded as burn-in. At this point the average standard deviation of split frequencies was below 0.01, the stationary phase of the log-likelihoods was reached and potential scale reduction factors equalled 1.0.

### Character state reconstruction

We performed character state reconstruction applying parsimony and ML criteria as implemented in Mesquite v2.71 [66], based on the consensus BI topology of LFG haplotypes. We examined three discrete characters, two of which are taxonomically important: long setae present or absent on the margins of the forewings and setae erect or adpressed on the hind femora, respectively (Table 1). The third character was reproductive mode (arrhenotoky vs. thelytoky). Additional sequences were included if multiple characters occurred for a given haplotype. For ML estimation the 'Markov k-state 1 parameter model' [67] and 'Asymmetrical Markov k-state 2 parameter model' were applied. Phylogenetic conservativeness was checked by testing for significant changes in the number of parsimonious steps in the original tree compared to trees with randomly reshuffled haplotypes. The root was supposed to be at equilibrium and transition rates were estimated by the program.

### Haplotype network

Based on the concatenated mtDNA sequences a 95% confidence statistical parsimony network was constructed for all LFG haplotypes using TCS [68]. Regarding sequences from thelytokous wasps, we assumed that additional individuals with the same microsatellite MLG (i.e., the same asexual line) also shared the same mitochondrial haplotype, and we included these unsequenced wasps in the haplotype frequency calculations. We refer to this extended haplotype set as 'extrapolated' data. This approach was justified as we confirmed that all of the 15 randomly selected pairs sharing a given microsatellite MLG (asexual samples from distant localities and different hosts, see above) were indeed consistently associated with identical mitochondrial haplotypes. Including the extrapolated data, 627 individuals were considered in the network (covering 118 out of 180 thelytokous MLG lineages, see Results).

### Genetic differentiation and microsatellite allele shared distances

Nuclear genetic differentiation was assessed using pairwise *F*_ST_. We partitioned the total sample progressively according to (*i*) reproductive mode and morphotype, (*ii*) reproductive mode and host aphid and (*iii*) reproductive mode, morphotype and host aphid. Only single MLG copies per morphotype and host species were included for each location [47,48]. We randomized MLGs among groups and locations within groups 1000 times for significance testing [45] and adjusted α-levels using the sequential Bonferroni correction. For comparisons defined under (*i*) and (*ii*) we also examined mtDNA differentiation. Estimates of *N*_ST _[69] and corresponding χ^2 ^significance tests (*K*_ST_) [70] were based on 1000 permutations [57].

As recommended by de Meeûs *et al*. [71,72], we further used factorial correspondence analyses (FCA) as implemented in GENETIX v4.03 to describe and illustrate overall genetic structure based on microsatellites [73]. Grouping was as in (*iii*) defined above. This method allows visualizing population (group) barycentres in a multidimensional space with as many dimensions as total number of alleles summed over all loci. Projection on the plane is defined by those axes which explain most of the total variation.

As a formal test of whether genetic differentiation-after geographic distance was accounted for-was stronger among samples from different hosts than among samples from the same host, we used partial Mantel tests [74] as implemented in Arlequin. Analyses were performed with nuclear and mitochondrial data. Single copies of each microsatellite MLG per host species and geographic origin were considered. We compared a matrix of pairwise genetic differentiation, that is *F*_ST _/(1-*F*_ST_), for microsatellites [75] and Kimura two-parameter distances for mtDNA sequences, with a binary matrix expressing whether two samples came from the same (0) or different (1) host, while controlling for geographic distance (log-transformed). Tests were based on 30'000 permutations.

Pairwise allele shared distances, *D_AS _*[76], were generated among all unique microsatellite MLGs using POPULATIONS [77]. We constructed a neighbour-joining (NJ) tree based on the distance matrix to assess overall genetic relationships irrespective of *a priori* groupings. Reproductive mode and morphotype characteristics (most common class for some ambiguous asexual MLGs) were manually plotted on the tree for visual inspection.

## Results

Our sampling effort retrieved more than 16.500 parasitoids of the LFG from seven major aphid host species across 15 European locations. To ensure independence of our data points, we generally considered only a single female genotype per parasitized aphid colony (our sampling unit). The genetic results are therefore based on a total of 911 diploid females. Their geographic origins, reproductive modes, host aphids and morphotypes are summarized in Table 2. Additional individuals were analyzed, either to identify the genotype of sporadic males found in clearly thelytokous samples, or to genetically confirm reproductive modes inferred from sample sex ratios (thelyokous samples typically consist of many females sharing the same MLG). These additional genotypes were not included in any analyses, but yielded interesting descriptive information. First, they showed that some thelytokous lines can produce males at low frequency (approx. 1: 300), confirming previous reports [8,9,78]. Genotyping identified most of these males as haploid, since they possessed single alleles at all loci the corresponding thelytokous MLGs were heterozygous for. This suggests that they arise by failed fusion of meiotic products during automixis. However, three samples contained diploid males. Second, we detected at least one triploid female each in a total of 18 out of 911 samples (approx. 2%).

### Distribution of reproductive modes and morphotypes

Overall, we collected many more thelytokous than sexual individuals of the LFG. The relative frequencies of reproductive modes varied among aphid hosts as well as among locations, which was reflected in significant effects of both factors on sample sex ratios (host: *F*_6, 751 _= 208.7, *P *< 0.001, location: *F*_14, 737 _= 20.1, *P *< 0.001). Sexuals were most common among samples from southern France, Switzerland and central Germany; elsewhere they were absent or exceedingly rare (Table 2 and Figure S1 [Additional file 1]). The significant host effect is due to all samples collected from *B. cardui *and more than half of the samples from *A. hederae *being sexual (Table 2). From all other host aphids, we obtained exclusively or almost exclusively thelytokous lines, the few exceptions mostly coming from southern France, where arrhenotoky was generally more common. Interestingly, the reproductive mode of wasps collected from *A. hederae *varied geographically. They were sexual in southern France, both reproductive modes occurred in central Europe, and only thelytokous lines were collected in eastern and northern Europe (Figure S2 [Additional file 2]). This was reflected in a near-significant location × host interaction on sample sex ratios (*F*_53, 684 _= 1.3, *P *= 0.083).

LFG morphotypes were non-randomly distributed across locations (Fisher's exact test, *P *< 0.001, Table 2). In addition, we found significant associations of morphotypes and the aphid hosts they parasitized (Fisher's exact test, *P *< 0.001). Although all three morphotypes could be collected from most of the aphids, their relative frequencies differed vastly (Table 2). The *Lfa *morphotype was the only one collected from *B. cardui *and the most common type on *A. f. fabae*, *A. urticata *and *A. hederae*. The *Lca *morphotype was the most abundant on *A. f. cirsiiacanthoides*, and the *Lco *morphotype was the only type found on *A. farinosa *and the most common type found on *A. ruborum*.

### Microsatellite variation

All nine microsatellite loci were successfully amplified in each sample and exhibited substantial polymorphism (11 alleles on average; Table 3). Because of the high frequency of thelytoky in all sampled areas, testing for deviations from linkage or Hardy-Weinberg equilibria was virtually pointless, yet analyses of purely sexual samples, restricted to single localities and hosts, provided no evidence for physical linkage or non-Mendelian inheritance in these markers, concordant with a previous report [39]. However, examining heterozygosity estimates of these loci after splitting individuals according to reproductive mode revealed some valuable insights (Table 3). Four loci (Lysi07, 15, 16 and 5a12) clearly behaved differently in thelytokous lines compared to sexuals. Thelytokous samples were either completely or almost completely homozygous at these loci, with corresponding *F*_IS _values of 1.0 (Lysi07) or close to 1.0 (Table 3). This was not the case for sexual samples.

**Table 3:** Genetic diversity estimates for the *Lysiphlebus fabarum *group.

			Microsatellite locus										
			
Test	Mode	Sample	Lysi03	Lysi05	Lysi06	Lysi07	Lysi08	Lysi13	Lysi15	Lysi16	Lysi5a12	Total	NRL
No. Alleles	Both	All	16	12	10	9	13	8	9	14	7	98	
	Sex	All	12	6	6	7	8	8	6	10	5	68	
	Asex	All	14	12	9	6	12	5	9	13	7	87	
	Asex	*Lfa*	12	11	8	3	12	5	9	12	7	78	
	Asex	*Lco*	12	8	8	6	7	5	7	9	6	68	
	Asex	*Lca*	6	6	7	3	6	5	7	9	4	53	
H_obs_	Sex	All	0.420	0.163	0.247	0.348	0.229	0.417	0.170	0.403	0.250	0.294	0.295
	Asex	*Lfa*	0.717	0.537	0.736	0.000	0.806	0.691	0.012	0.004	0.002	0.389	0.697
	Asex	*Lco*	0.723	0.547	0.679	0.000	0.170	0.533	0.010	0.010	0.018	0.299	0.531
	Asex	*Lca*	0.884	0.351	0.927	0.000	0.787	0.739	0.014	0.018	0.004	0.414	0.738
*F*_IS_	Sex	All	**0.393**	0.048	**0.222**	**0.448**	**0.368**	**0.389**	**0.397**	**0.325**	**0.238**	**0.344**	**0.338**
	Asex	*Lfa*	-0.073	-0.015	-0.108	**1.000**	-0.226	-0.074	**0.977**	**0.993**	**0.988**	**0.312**	-0.100
	Asex	*Lco*	0.021	**0.186**	-0.018	**1.000**	**0.419**	-0.129	**0.981**	**0.982**	**0.958**	**0.471**	**0.080**
	Asex	*Lca*	-0.436	0.032	-0.336	**1.000**	-0.269	-0.383	**0.974**	**0.945**	**0.955**	**0.092**	-0.296
*F*_ST_	Sex	All	**0.097**	**0.154**	**0.118**	**0.262**	**0.248**	**0.115**	**0.257**	**0.195**	**0.297**	**0.203**	**0.151**
	Asex	*Lfa*	**0.109**	**0.088**	**0.102**	**0.368**	**0.060**	**0.091**	**0.080**	**0.077**	**0.205**	**0.102**	**0.091**
	Asex	*Lco*	**0.119**	**0.082**	**0.095**	**0.161**	**0.136**	**0.046**	**0.131**	**0.169**	**0.192**	**0.127**	**0.096**
	Asex	*Lca*	**0.141**	**0.212**	**0.144**	**0.158**	**0.100**	**0.206**	**0.375**	**0.273**	**0.517**	**0.241**	**0.160**

Number of alleles, observed heterozygosities, inbreeding coefficients (*F*_IS_), and genetic differentiation across sampling locations (*F*_ST_) are shown for each microsatellite marker individually, across the total marker set (total), as well as all markers excluding Lysi07 and recombining Lysi15, 16 and 5a12 (NRL, non recombining loci only). Estimates are presented for the total sample (all), different reproductive modes (definitions see Table 2) and for individual morphotypes of the pool of asexuals separately (see Table 1). Bold cells indicate significant homozygote excess and differentiation among populations, respectively.

Complete homozygosity of asexuals at locus Lysi07 is readily explained by the known linkage of this locus to the genomic region responsible for reproductive mode variation in *Lysiphlebus *[17]. The vast majority of thelytokous females from all three morphotypes were homozygous for a single allele at this locus (allele 183), consistent with a previous study reporting perfect linkage of this allele to a recessive, thelytoky-inducing genetic factor in *Lfa *[17]. Here, we found five additional alleles in total associated with thelytoky. These alleles mainly occurred in lineages belonging to the *Lco *morphotype sampled from *A. farinosa*, and they always occurred in the homozygous state as well. Because of its association with reproductive mode, this locus was excluded from all population genetic analysis.

Loci Lysi15, 16 and 5a12 are neither associated with reproductive mode nor linked to Lysi07 [see also [39]]. For these three loci, near-complete homozygosity in asexuals must result from central fusion automixis [15]. This cytological mechanism of diploidy restoration retains heterozygosity in non-recombining regions of the genome and between centromeres and chiasmata when recombination takes place, but leads to 50% homozygous offspring in regions distal of chiasmata [10]. Indeed, when newly generated asexual lineages [17] were repeatedly re-genotyped, a progressive loss of heterozygosity at the same three loci was observed, whereas original genotypes at loci Lysi03, 05, 06, 08 and 13 remained unchanged (CS & CV, unpubl.). This suggests that these five loci are located close to centromeres or in other non-recombining genomic regions, such as chromosomal inversions. However, at least in paracentric inversions heterozygosity can also be lost under central fusion automixis if crossovers occur between the centromere and the inversion. We refer to loci Lysi03, 05, 06 and 13 as non-recombining loci (NRL), and to loci Lysi15, 16 and 5a12 as recombining loci (RL).

At the NRL, heterozygosity tended to be substantially higher in asexual samples than in sexuals (Table 3). Sexual samples generally exhibited heterozygote deficits, which was reflected in positive *F*_IS _values that were significantly larger than zero for all but one locus (Table 3). To some extent this presumably reflects a Wahlund effect from pooling individuals collected from different host aphids on which parasitoids may be specialized, yet it may also indicate the strong philopatry characteristic of *Lysiphlebus*, resulting in local inbreeding [79].

*F*_ST _values were significant for sexual and thelytokous samples across all loci, indicating substantial genetic differentiation of *Lysiphlebus *parasitoids among locations. To what extent this reflects geographic or ecological (e.g. host specialization) limits to gene flow is addressed in more detail below.

### Nuclear genotypic diversity

The high allelic diversity of the microsatellite loci provided sufficient resolution to conclude that individuals with the same MLG were members of the same thelytokous line. The probabilities of repeatedly observed MLGs to be produced by independent sexual events were very low (*p*_sex _< 0.0015). Accordingly, all 205 individuals determined as sexual had unique MLGs, but among the 706 individuals determined as thelytokous, only 180 different MLGs could be distinguished (Table 2). Therefore, the genotypic diversity estimated as *R *was higher at locations containing sexuals than in areas from which only thelytokous parasitoids of the LFG were found (*F*_1, 12 _= 8.986, *P *= 0.011; see Table 2).

A total of 77 different MLGs were collected more than once. Of those, 27 were collected at multiple locations and 43 were collected from multiple host species. Sixteen MLGs exhibited morphological variation to the extent that individuals with the same genotype were classified as different morphotypes. Table 4 summarizes the distribution of all MLGs that were detected at least 10 times and reveals that different thelytokous *Lysiphlebus *lines vary strongly in their degree of host specialization. The two most common lines (MLG *i *and *ii*), for example, seem to be widespread generalists. They occurred 43 times across ten and 34 times across eight locations, respectively, and parasitized almost all considered host aphids (Table 4). The third most common line (MLG *iii*), on the other hand, occurred 30 times across five locations, but was exclusively collected from *A. ruborum*. Similarly, this table identifies lines that appear to be specialized on *A. f. cirsiiacanthoides *(MLGs *viii*, *x*, *xxi*), *A. hederae *(MLGs *iv*, *v*, *xix*) and several additional *A. ruborum *specialists (MLGs *vi*, *vii*, *ix*, *xi*, *xvii*, *xxiii*). Furthermore, morphotype is associated with host specialization. Although these patterns are not exclusive, they are consistent with the highly significant association of host aphid and morphotype in our complete sample of parasitoids from the LFG, as reported above.

**Table 4:** Collection details of the most abundant thelytokous lineages of the *Lysiphlebus fabarum *group.

	Host							Morphotype			
				
Asex MLG	*Aff*	*Afc*	*Aur*	*Ahe*	*Aru*	*Asp*	Areas	*Lfa*	*Lco*	*Lca*	total
*i*	16	10	7	7	1	2	10	43	-	-	43
*ii*	10	11	6	-	1	6	8	1	-	33	34
*iii*	-	-	-	-	30	-	5	22	8	-	30
*iv*	1	1	-	26	-	-	2	28	-	-	28
*v*	2	-	-	24	-	-	1	26	-	-	26
*vi*	-	-	1	3	19	-	5	-	23	-	23
*vii*	-	2	1	1	18	-	3	1	21	-	22
*viii*	-	14	2	2	-	3	3	-	-	21	21
*ix*	-	-	-	1	19	1	2	3	18	-	21
*x*	1	12	2	-	1	3	3	-	1	18	19
*xi*	-	-	-	-	16	1	2	-	17	-	17
*xii*	7	8	-	-	-	-	1	2	-	13	15
*xiii*	5	4	-	4	-	1	2	1	1	12	14
*xiv*	7	5	-	2	-	-	1	-	-	14	14
*xv*	8	1	-	3	1	-	2	1	12	-	13
*xvi*	4	4	1	1	-	3	3	13	-	-	13
*xvii*	-	-	-	1	12	-	3	4	9	-	13
*xviii*	4	-	-	8	-	-	2	12	-	-	12
*xix*	-	1	-	11	-	-	2	12	-	-	12
*xx*	6	2	2	-	-	1	1	11	-	-	11
*xxi*	-	10	-	1	-	-	1	-	-	11	11
*xxii*	8	-	-	-	2	-	1	10	-	-	10
*xxiii*	-	-	-	-	10	-	2	10	-	-	10

Records for diverse hosts (see Table 2), corresponding morphotypes (see Table. 1) and the number of sampling locations where they were found (areas) are indicated for all thelytokous microsatellite multilocus genotypes (MLG) detected at least ten times (total).

### Mitochondrial sequence diversity

**Figure 1:**
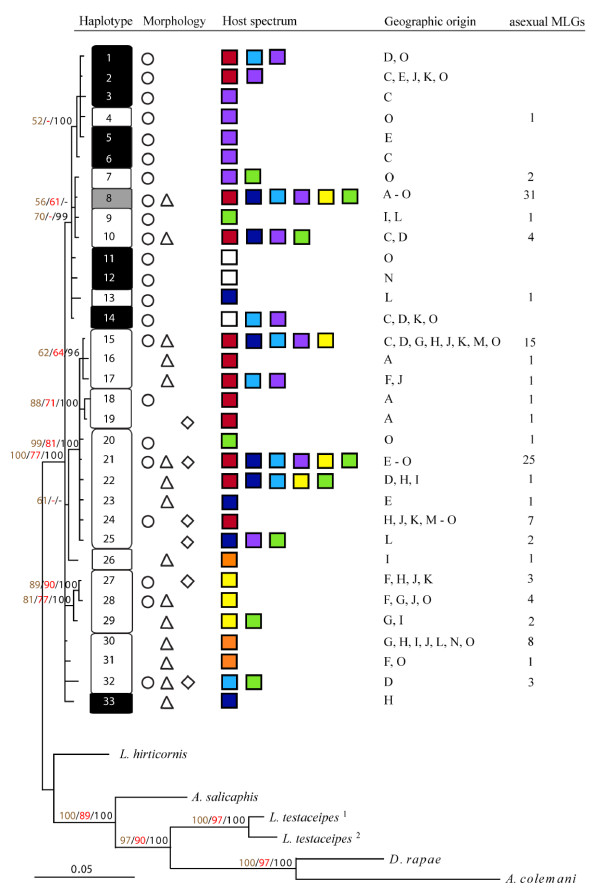
**Bayesian phylogram derived from the combined COI and ATP6 data sets**. Support is indicated on the nodes, i.e., bootstrap values for maximum parsimony (brown) and maximum likelihood (red) and Bayesian posterior probability (black) (only bootstrap values above 50% and posterior probabilities above 90% are shown). The scale bar indicates substitutions per site. Haplotypes of the *Lysiphlebus fabarum *group parasitoids are coded as numbers on the tips of each branch. Reproductive modes associated with individual haplotypes are indicated as follows: purely sexual, white numbers on black; purely asexual, black numbers on white; both reproductive modes, black numbers on grey. Morphotypes (see Table 1) are represented as follows: circles, *Lfa*; triangles, *Lco*; squares, *Lca*. Different colours correspond to diverse host origins as follows: *Aphis fabae fabae *(green), *A. f. cirsiacanthoidis *(dark blue), *A. urticata *(yellow), *A. hederae *(purple), *A. ruborum *(red), *A. farinosa *(orange), *Aphis sp*. (light blue) and *Brachycaudus cardui *(white). Records for diverse locations (see Table 2) indicate geographic ranges of individual haplotypes. The number of unique microsatellite multilocus genotypes of asexual lineages is indicated for corresponding haplotypes. Details concerning outgroup taxa see Table S1 [see Additional file 3].

COI and ATP6 sequences were obtained from a representative subsample of 170 LFG specimens and ten specimens from six outgroup taxa. Haplotypes have been deposited in GenBank, accession numbers and details are given in Tables S1 and S2 [Additional files 3 &4]. The total alignment of 1302 bp contained 267 variable sites, of which 146 were parsimony informative. Within the LFG, 61 sites were variable (25 parsimony informative, 12 for COI and 13 for ATP6), and only eight substitutions were non-synonymous (two in COI, six in ATP6). Based on the concatenated genes, LFG parasitoids comprised 33 distinct haplotypes. Phylogenetic trees constructed with BI, ML and MP were highly congruent, the consensus BI topology is depicted in Figure 1. The split between the LFG and the outgroups, as well as all splits among outgroup taxa were well supported and based on 3.2% or more sequence divergence. Yet, within the LFG, tree topology was very shallow and obtained poor statistical support (Figure 1). Haplotype divergence did not exceed 1.32%, which was less than that observed between the two haplotypes of the outgroup taxon *L. testaceipes *(1.61%). The limited mtDNA variation in the LFG was to some extent associated with reproductive mode. Only the common haplotype 8 was shared by thelytokous and sexual wasps (albeit present in only one sexual individual), and haplotypes associated with sexual wasps were highly aggregated in one major branch of the LFG tree (Figure 1). Indeed, the character state analysis detected a significant phylogenetic signal in the distribution of reproductive modes (*P *< 0.01). Nevertheless, few haplotypes associated with thelytoky fell into the same group as most haplotypes from sexuals. The more common haplotypes were found in many (up to 31) different thelytokous MLGs (Figure 1).

**Figure 2:**
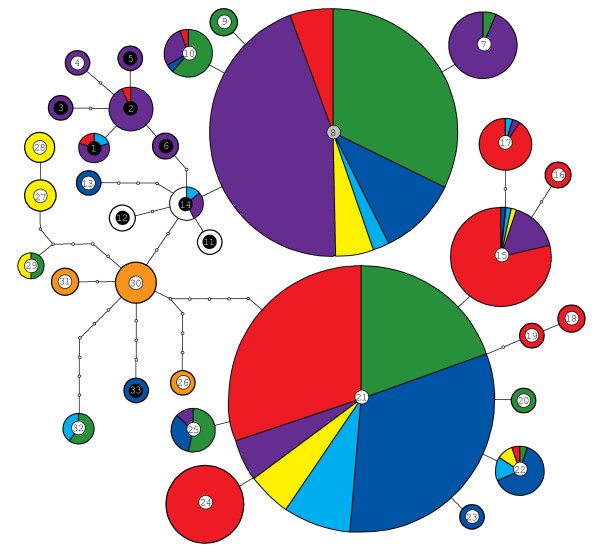
**Haplotype network based on the combined COI and ATP6 data sets**. Each haplotype is represented by a circle with approximately scaled areas indicating the numbers of samples possessing a given haplotype. Colours indicate proportions of samples associated with divergent host species as detailed in Figure 1. Haplotypes are connected by lines corresponding to single nucleotide substitutions, small colourless circles denote 'missing haplotype' nodes. Labels for individual haplotypes and associated reproductive modes are the same as detailed in Figure 1 and placed within the centre of each circle area.

Associations between mitochondrial haplotypes and host use of LFG parasitoids were weak (Figure 1) and are best reflected in the haplotype network depicted in Figure 2. Wasps collected from *A. hederae *mostly possessed the common haplotype 8 or closely related ones, wasps from *A. ruborum *mostly had the common haplotype 21 or very similar ones, and all wasps from *B. cardui *shared very similar haplotypes (Figure 2). Yet, Figure 2 also shows that the more abundant haplotypes were found in wasps collected from almost all host aphids.

There was no consistent pattern of haplotype association with morphotypes. Eight haplotypes comprised individuals belonging to two or even all three different morphotypes (Figure 1). Accordingly, the character state analysis revealed that neither the presence or absence of long setae on the margins of the forewing nor the orientation of setae on the hind femora (Table 1) were phylogenetically conservative characters (*P *> 0.05).

### Genetic differentiation

We evaluated genetic differentiation based on *F*_ST _(microsatellites) and *N*_ST _(mtDNA) at different levels of population subdivision to investigate the structuring of genetic variation in the LFG with respect to reproductive mode, morphotype and host use. When individuals were grouped according to their reproductive mode and morphotype (Table 5), nuclear and mitochondrial differentiation was significant between all groups, but strongest between the sexuals (all *Lfa*) and the thelytokous groups of all three morphotypes. There was also substantial differentiation among sampling locations within all groups (diagonal in Table 5).

**Table 5:** Genetic differentiation among morphotypes within reproductive modes of the *Lysiphlebus fabarum *group.

			Sex	Asex		
				
Mode	Morph		*Lfa*	*Lfa*	*Lco*	*Lca*
		Area	8	14	15	12
		MLG	202	182	87	76
		Seq	36	95	61	35
Sex	*Lfa*		***.180***	**.188**	**.254**	**.224**
Asex	*Lfa*		**.409**	***.099***	**.072**	**.083**
	*Lco*		**.578**	**.111**	***.123***	**.131**
	*Lca*		**.806**	**.375**	**.152**	***.242***

Nuclear genetic differentiation (*F*_ST_) between groups (above diagonal, definitions see Tables 1 and 2) and within groups among geographic locations (diagonal, in italics) is indicated, as well as mitochondrial sequence differentiation, *N*_ST _(below diagonal). Overall numbers of areas, microsatellite multilocus genotypes (MLG) and mtDNA sequences (Seq) are detailed. Bold values indicate significant differentiation.

When parasitoids were grouped according to reproductive mode and host aphid, ignoring morphology (Table 6), nuclear and mitochondrial differentiation was also significant for most pairwise comparisons. Again, differentiation was much stronger between sexual and thelytokous wasps from all hosts than between thelytokous parasitoids from different hosts. The only comparison between reproductive modes with a comparatively low *F*_ST _value < 0.2 concerned wasps from *A. hederae*, the only aphid on which sexual and asexual *Lysiphlebus *overlap strongly. Table 6 also shows that the two groups of sexuals from *A. hederae *and *B. cardui *are clearly differentiated (*F*_ST _= 0.317). Among thelyokous wasps, those collected from *A. farinosa *and *A. ruborum *were each strongly differentiated from the other host-associated groups (all *F*_ST _> 0.12 and > 0.08, respectively). Asexual parasitoids from *A. f. fabae*, *A. f. cirsiiacanthoides *and *A. urticata*, on the other hand, were not significantly differentiated from each other, and they were also closely related to asexuals from *A. hederae *(Table 6). Differentiation among locations was significant for all groups except for wasps collected from *A. urticata*. Mitochondrial differentiation provided a similar picture overall.

**Table 6:** Genetic differentiation among host-affiliated samples within reproductive modes of the *Lysiphlebus fabarum *group.

			Sex		Asex					
				
Mode	Host		*Ahe*	*Bca*	*Aff*	*Afc*	*Aur*	*Ahe*	*Aru*	*Afa*
		Area	7	4	15	11	9	13	13	8
		MLG	180	14	74	54	31	58	74	22
		Seq	24	7	53	33	26	39	54	12
Sex	*Ahe*		***.208***	**.317**	**.214**	**.235**	**.234**	**.129**	**.298**	**.341**
	*Bca*		**.675**	***.108***	**.331**	**.366**	**.387**	**.300**	**.347**	**.495**
Asex	*Aff*		**.527**	**.302**	***.101***	.002	.016	**.022**	**.084**	**.132**
	*Afc*		**.650**	**.551**	**.141**	***.102***	.023	**.034**	**.102**	**.127**
	*Aur*		**.564**	**.437**	**.154**	**.146**	*-.026*	**.033**	**.101**	**.160**
	*Ahe*		**.534**	**.310**	-.001	**.150**	**.178**	***.105***	**.090**	**.138**
	*Aru*		**.785**	**.728**	**.361**	**.079**	**.268**	**.367**	***.111***	**.167**
	*Afa*		**.808**	**.700**	**.372**	**.462**	**.276**	**.411**	**.628**	***.116***

Nuclear genetic differentiation (*F*_ST_) between host groups (above diagonal, definitions see Table 2) and within groups among geographic areas (diagonal, in italics) is indicated, as well as mitochondrial sequence differentiation, *N*_ST _(below diagonal). Overall numbers of areas, microsatellite multilocus genotypes (MLG) and mtDNA sequences (Seq) are detailed. Bold values indicate significant differentiation.

Grouping parasitoids according to reproductive mode, morphotype and host aphid (microsatellite data only; Table S3 [Additional file 5]) overall confirmed the results mentioned above. In particular, differentiation between sexual and asexual *Lfa *morphotypes on *A. hederae *was comparatively low. Thelytokous *Lfa *morphotypes from *A. ruborum *were clearly differentiated from *Lfa *morphotypes from other hosts, but less differentiated from *Lco *morphotypes from the same host. *Lco *morphotypes from *A. farinosa *were strongly differentiated from all other groups, including other *Lco *morphotypes. On the other hand, thelytokous *Lca *morphotypes from various host aphids represented a relatively homogeneous group. A small sample of sexuals with *Lco *morphotype collected from *A. f. cirsiiacantoides *appeared to be strongly differentiated from other sexuals as well as all thelytokous groups.

**Figure 3:**
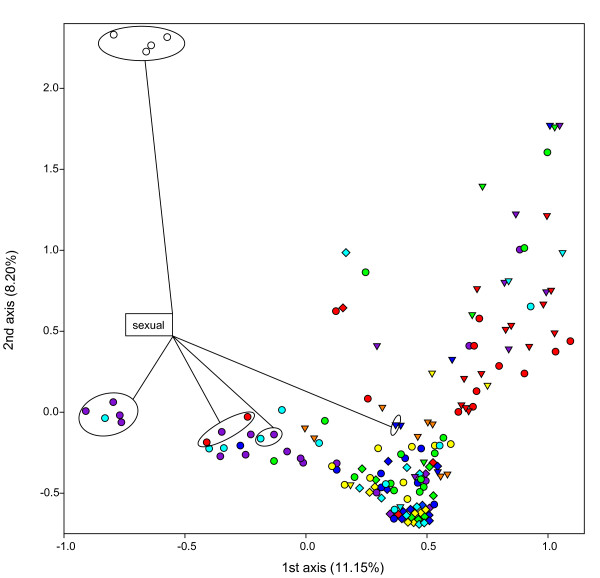
**Factorial correspondence analysis based on microsatellite data depicting populations of *Lysiphlebus fabarum *group parasitoids**. The sample was hierarchically grouped into reproductive mode, morphotype (see Table 1), host origin and geographic location (excluding replicate MLGs) (see Table S3 [Additional file 5]). The same symbols for morphotypes and colours for host species as detailed in Figure 1 are used in combination for samples corresponding to a given geographic origin. The 'centers of gravity' of these populations are projected on the plane defined by the first two axes (corresponding percentages of explained total inertia are indicated). Sexual populations are highlighted while all other data points refer to asexual populations.

The FCA largely recapitulated observations based on nuclear differentiation and allowed a graphical illustration of relations among groups of parasitoids as defined in Table S3 [Additional file 5] (Figure 3). As suggested by their strong differentiation, sexual wasps collected from *B. cardui *formed a clearly separate group. Points representing sexual wasps from *Aphis *hosts also clustered, although a geographic component became evident: central and southern European sexuals formed slightly separate clusters, with the latter showing stronger overlap with thelytokous groups, especially those from *A. hederae*. Only the unusual group of sexuals with *Lco *morphotype fell amidst the asexuals. Figure 3 further illustrates that the vast majority of thelytokous *Lca *morphotypes formed a very narrow cluster. Thelytokous *Lfa *and *Lco *morphotypes, however, were very heterogeneous, although some structure reflecting host use was evident, such as the clustering of most points representing wasps from *A. ruborum*, independent of morphotype, or the close association of points representing *Lco *from *A. farinosa *(Figure 3). Similarly, the majority of groups from *A. f. fabae*, *A. f. cirsiiacanthoidis *and *A. urticata *formed a loose cluster irrespective of morphotype.

We further compared matrices of pairwise nuclear genetic differentiation or mtDNA distance with matrices of pairwise geographic distance as well as matrices expressing whether samples came from the same or different host species in a partial Mantel test (Table 7). Pairwise nuclear genetic differentiation and mtDNA distances were significantly larger for samples collected from different aphid hosts, most apparent after correction for spatial separation. We detected a significant overall isolation by distance within host associated groups at nuclear markers, but not for mtDNA. Yet, we found no significant correlation between matrices of nuclear genetic differentiation and mtDNA distances corrected for either host species or geographic distance (for all correlations *P *> 0.15, not shown).

**Table 7:** Partial Mantel tests for partitioning of nuclear and mitochondrial genetic variation in *Lysiphlebus fabarum *group parasitoids.

	Microsatellites		mt DNA	
		
Correlation of genetic distance with	*r*	*P*	*r*	*P*
Host species	0.130	< 0.001	0.052	0.008
Geography	0.099	< 0.001	0.009	0.347
Host species controlled for geography	0.140	< 0.001	0.053	0.006
Geography controlled for host species	0.111	< 0.001	0.014	0.283

### Individual genetic relationships

**Figure 4:**
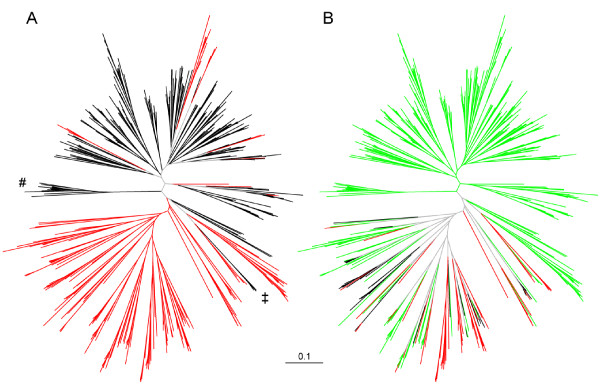
**Neighbour-joining cladogram of 385 unique microsatellite multilocus genotypes of the *Lysiphlebus fabarum *group parasitoids based on allele shared distances**. A. Relationship among sexual (black) and asexual (red) genotypes and lineages, respectively. ^# ^indicate the clade of parasitoids *ex Brachycaudus*; ^‡ ^indicate sexual samples of the *Lco *morphotype (see Table 1). B. Genetic relationship among morphotypes: *Lfa *(green), *Lco *(red) and *Lca *(black).

The (incomplete) genetic isolation between sexual and thelytokous parasitoids of the LFG is most readily seen in the NJ tree based on allele-shared distances (*D*_AS_) of all 385 unique MLGs (Figure 4A). There is a strong separation between reproductive modes, but a few MLGs belonging to asexual wasps are interspersed in the predominantly sexual branches of the tree. All of these asexuals were collected from *A. hederae*. Sexuals from *B. cardui *occupy a separate branch at the base of most other sexuals, and the sexuals with *Lco *morphotype are separate from other sexuals within a branch composed largely of thelytokous MLGs.

The same tree color-coded for morphotypes (Figure 4B) confirms that except for the three individuals with *Lco *morphotype, all sexual wasps belonged to the *Lfa *morphotype. The tree further shows that the morphotypes of thelytokous MLGs are widely mixed. Morphology is thus a poor predictor of genetic relatedness in thelytokous parasitoids of the LFG.

## Discussion

This comprehensive analysis of sexual and asexual parasitoids of the LFG using population genetic and phylogenetic approaches provided the following main insights: (1) These aphid parasitoids represent a closely related group in which thelytoky predominates, and in which the occurrence of sexual reproduction shows a strong pattern of host association, as well as geographic variation. (2) Reproductive modes tend to be aggregated on the mitochondrial tree, as previously reported [8], albeit with important exceptions. (3) Nuclear genetic differentiation between reproductive modes was generally strong, but lowest for wasps collected from *A. hederae*, the only host on which arrhenotokous and thelytokous parasitoids commonly co-occur. (4) Nuclear genotypic diversity is very high in asexuals, indicating frequent transitions to asexuality and/or the frequent occurrence of 'cryptic sex'. (5) Nuclear differentiation among parasitoids collected from different aphids indicates host specialization. (6) Characters used traditionally in the taxonomy of LFG parasitoids poorly reflect their genetic relationships. We discuss these findings in more detail below.

### Genetic relationships between reproductive modes

The incomplete genetic separation at nuclear and mitochondrial loci clearly indicates that the gene pools of sexual and asexual parasitoids of the LFG are not fully independent (Figures 1 &4A). But how does gene flow take place between reproductive modes?

One feasible route is the experimentally demonstrated formation of new thelytokous lines via males introgressing the recessive thelytoky-inducing genetic factor into sexual populations [17]. This field survey indeed provides evidence for contagious parthenogenesis: Few thelytokous mitochondrial haplotypes fell into a mainly sexual clade (Figure 1), and corresponding individuals were identified as the few asexual microsatellite MLGs interspersed in the large sexual clade of the NJ tree (Figure 4A). This supports the view that recessive thelytoky requires inbreeding after introgression into sexual lineages to be expressed [17]. However, if this route was very common, it should rapidly erode any association of mitochondrial variation and reproductive mode. Hence, contagious parthenogenesis is unlikely to be the only or most important route of gene flow between reproductive modes.

An alternative route is introgression from sexuals into asexual lineages, as proposed previously [8]. If thelytokous females mate with males from sexual populations, they may include paternal alleles rather than complete meiotic parthenogenesis [15]. For the parasitoid *Venturia canescens*, which also exhibits central fusion automixis [16], this route of introgression has been documented and shown to strongly affect the overall genetic relationships among sexual and asexual populations [80,81]. We suspect such genetic exchange to also occur in LFG parasitoids (Table 6 & Figure 3), as males from sexual lines readily mated with thelytokous females in the laboratory (CS, pers. obs.). However, the recessiveness of thelytoky [17] poses a major challenge to predict the effective direction of gene flow between reproductive modes and evolutionary outcomes in general. Both pathways of introgression would increase the frequency of the recessive thelytoky-inducing factor in sexual populations and may thus operate synergistically in elevating asexual genotypic diversity, but they cannot explain the strong genetic differentiation between reproductive modes (Figures 1 &4A).

A third route reconciling the high genotypic diversity in asexuals with the still substantial genetic differentiation from sexuals is cryptic sex within thelytokous populations of the LFG. It also relies on the occasional production of males by thelytokous lines (i.e., male carriers of a thelytoky-inducing allele [17]). In most instances, the only females these males will encounter are thelytokous females. Occasional sperm incorporation via fertilization rather than automictic diploidy restoration in thelytokous females [15,81] would then result in genetic exchange between individuals of thelytokous origin. This third mechanism of covert sex can readily maintain a high genotypic diversity in asexual populations without gene flow from sexuals, thereby allowing the buildup of genetic differentiation between the two groups, consistent with our observations and previously reported patterns [8].

Probably all three mechanisms of genetic exchange play a role in shaping the overall genetic architecture of the sexual-asexual LFG complex, but their relative importance is likely to differ regionally. In mixed populations and on shared hosts there appears to be ample opportunity for gene flow between reproductive modes. In purely thelytokous populations the third mechanism may be the only one available. All pathways rely on males produced by thelytokous lines being functional [17] and on thelytokous females being able to use sperm occasionally, for which central fusion automixis seems to be especially eligible [15,81,82]. This indicates that this sexual-asexual complex is an evolutionarily young system, which is also supported by the shallow mitochondrial genealogy of LFG parasitoids.

The rare but geographically widespread detection of triploid females was interesting. Such females may originate either from thelytokous females occasionally fertilizing their diploid eggs with haploid sperm, or else from sexual females fertilizing their haploid eggs with diploid sperm produced by diploid males [83]. The fact that in our survey, triploid females were detected exclusively in all-females samples, suggests that they were produced by the former route. Nevertheless, diploid males were also detected in three cases. Diploid males are known to occur under inbreeding in sexual Hymenopterans with complementary sex determination (CSD, reviewed in [84]). Inbreeding increases the probability of homozygosity at the CSD locus (in species with single locus CSD) or at all CSD loci, respectively (in species with multilocus CSD), which results in male development of diploid offspring. Diploid males produced by thelytokous lineages highlight an interesting interaction that can occur between automixis and sex determination in thelytokous Hymenopterans (discussed in [85]). Just like inbreeding in sexuals, automixis in asexuals increases offspring homozygosity, which in turn might result in thelytokous females producing some proportion of diploid males if the CSD loci are situated in recombining regions of the genome. We have preliminary evidence that the sex determination system in LFG parasitoids corresponds to multilocus CSD, and that laboratory-generated thelytokous lines may indeed produce some diploid male offspring that are functional and able to sire daughters (CS & CV, unpublished data). In principle, such males could be efficient vehicles to spread the recessive, thelytoky-inducing allele into sexual populations, but our field data show that male production by thelytokous LFG parasitoids is generally rare, and diploid male production even rarer than haploid male production. Note that the mechanism leading to the production of haploid males (occasional failure of central fusion) is different from the mechanism we propose for diploid male production (homozygosity at CSD loci). Thus, it appears that thelytokous lineages producing a noteworthy proportion of diploid male offspring are disfavoured by selection, as expected [85]. Clearly, the interplay between the genetic determination of thelytoky and the sex determination system as well as the role of triploids in this interesting group of parasitoids deserves further research.

### Host associations

Apart from the observed differentiation between reproductive modes, nuclear markers also indicated limitations to gene flow between parasitoids collected from different aphids, i.e., host-associated differentiation (HAD). This was most obvious for the exclusively sexual wasps collected from *B. cardui*, which were clearly differentiated from all asexual groups as well as other sexuals. Their separate status was recognized in a previous genetic investigation [8] and is further supported by their possession of characteristic cuticular hydrocarbon profiles [36]. Yet, no clear divergence is evident from mitochondrial data. Haplotypes found in wasps from *B. cardui *were either shared with or closely related to wasps collected from *Aphis *hosts (Figures 1 &2). This suggest a recent acquisition of *B. cardui *as a host. Further, the close similarity of *B. cardui*-attacking wasps from geographically distant locations indicates that this host switch did not occur independently in different regions (Figures 3 &4A).

Other well-differentiated groups were the thelytokous wasps collected from *A. farinosa *and *A. ruborum*, and the sexual wasps from *A. hederae *(Table 6). This strongly indicates the evolution of host specialization in the LFG. Host fidelity due to imprinting during development is known from *Aphidius *parasitoids [86-88]. They prefer the same aphid-host plant assemblages on which they developed for oviposition, presumably based on olfactory cues [89]. *Lysiphlebus *wasps also tend to exhibit better performance after conditioning [90] and they mate and oviposit very soon after emergence on or close by their natal patch [91]. Genetic exchange between parasitoids associated with different hosts will be further restricted if higher fitness on a particular aphid host entails reduced performance on others. There is evidence for such trade-offs from host switch and selection experiments in other aphid parasitoids [88,92]. Indeed, on certain plants, mixed host colonies of *B. cardui *and *A. f. cirsiiacanthoidis *are common, suggesting that HAD in LFG parasitoids is held up despite ample opportunities for interbreeding [93,94]. Additional indirect evidence for trade-offs in host performance was gained by establishing laboratory cultures of field-collected wasps on *A. f. fabae*. Establishing wasps collected from *A. f. fabae *or *A. f. cirsiiacanthoides *is generally easy, establishing wasps from *A. hederae*, *A. urticata *and *A. ruborum *is more difficult but possible, and establishing wasps from *A. farinosa *is near-impossible ([8], CS & CV, unpubl.).

Regarding the close relationships among sexual *Lysiphlebus *affiliated with different *Aphis *hosts in southern France (Figure 3), little gene flow may be sufficient to erode patterns of HAD at neutral markers in sexual parasitoids [31,92]. Yet, many of the common thelytokous MLGs had very restricted host ranges (Table 4). This indicates strong specialization which may primarily emerge in thelytokous LFG parasitoids, because a genotype that is particularly well adapted to a certain host will not be broken up by recombination. Possibly, the strong host specialization of certain genotypes is related to *Lysiphlebus*' strategy of chemical camouflage to avoid detection by tending ants [36], which might only work on a single aphid host. Nevertheless, Table 4 also shows that strongly restricted host ranges are by no means an unavoidable evolutionary outcome. Some of the most common thelytokous MLGs were collected from various hosts. The remarkable host range variation of different thelytokous lineages in the LFG clearly deserves further investigation.

### Phylogeography and geographic parthenogenesis

We observed a geographic signal in the distribution of sexual and asexual populations of *Lysiphlebus *associated with *Aphis *hosts, apparently reflecting geographic parthenogenesis [95,96]: on most hosts in northern and eastern Europe thelytokous populations dominate, while sexuals are prevalent in southern France, where they use a large host range. The fact that central European sexuals associated with *A. hederae *represent a subset of the haplotypic diversity of southern populations (Figure 1) suggests that glacial refuges may have been located in Mediterranean or Iberian areas. Range expansion from these regions is also indicated in gallwasps, for example [97]. Assuming that both reproductive modes were already co-residing in former refuges, higher colonization abilities of asexuals [98] with subsequent monopolization of the habitats [99] might have influenced this pattern. Yet, present ecological forces could be relevant as well. Shorter growth seasons in more temperate regions, coupled with 'boom-and-bust' dynamics of aphid hosts may favour asexuals in balancing frequent local extinction events with stochastic recolonization [100]. Indeed, populations associated with *B. cardui *indicate that in the absence of asexual competitors, sexual parasitoids prevail.

However, the phylogenetic aggregation of reproductive modes and low levels of asexual haplotype diversity in southern France (Figure 1) suggest that many thelytokous lineages residing in northern and eastern Europe originally stem from other geographic sources, not considered here. A similar pattern is indicated in *V. canescens*, where sexual populations are only known from southern France [80]. Indeed, LFG parasitoids have been also reported from the Balkans, Anatolia and the Near East [8,23,93,101-104], including morphologically variable sexual populations from various hosts. Some of these areas were shown to represent major hot spots of genetic diversity, e.g. in gallwasps [105]. In that group, there is evidence that south-western Europe was colonized from Iberian refuges after the last ice age while other European populations could be traced back to south-eastern refuges [97,105]. Assuming that *Lysiphlebus *exhibited similar range expansions, we strongly recommend including samples from south-eastern areas in future assays to allow more detailed inferences on phylogeographic patterns and the evolutionary history of reproductive modes.

### Morphological variation

The morphological variation in LFG parasitoids was certainly informative ecologically. The three morphotypes (Table 1) tended to be associated with certain host aphids, although these associations were rarely exclusive. Genetic analyses showed, however, that morphological variation carried little phylogenetic information, as previously suggested by Belshaw *et al*. [8]. Thelytokous parasitoids of different morphotypes were widely mixed in the mitochondrial genealogy with some haplotypes found across all three morphotypes (Figure 1). Moreover, morphotypes were strongly admixed in the NJ-tree based on microsatellite genotypes (Figure 4B). On the other hand, some host associated groups of the same morphotype displayed strong nuclear divergence (Table S3 [Additional file 5]).

It appears that to a limited extent, the morphological characters used in LFG taxonomy exhibited variable expression. We had rare cases in which different individuals from the same thelytokous MLG were classified as different morphotypes (Table 4), suggesting some degree of plasticity in these traits. Generally, however, the characters underlying morphotype definitions breed true and are stably expressed over many generations in laboratory cultures (CS & CV, pers. obs.). Hence, it is likely that morphological differences among asexual lineages represent 'frozen' variation that was captured when they split from sexual, morphologically variable, source populations. Indeed, crossing experiments using sexual *Lfa *and *Lco *morphotypes indicate that the relevant traits are under nuclear genetic control [see [93]]. Thus, the observed variation within thelytokous populations, which may well be ecologically relevant, can also be expected to be fed by genetic exchange resulting from 'cryptic sex' as described above (Figure 4B).

### Implications for taxonomy

According to current taxonomy, the three distinguishable morphotypes (Table 1) are treated as distinct species within the LFG [22,24,106-109]. However, taxonomists are well aware of their problematic status [8,22]. In accordance with Belshaw *et al*. [8], we conclude that these species boundaries cannot be upheld. This is for two main reasons: First, the morphological characters used for species definitions are not phylogenetically conservative. Second, the mitochondrial sequence divergence of no more than 1.54% at COI across the whole LFG is well within what is considered a normal level of within-species variability in molecular taxonomy [51,110]. Only the group of sexual wasps collected from *B. cardui *might well deserve a separate taxonomic status, as already proposed by Starý [22]. However, this would solely be based on their nuclear differentiation and their specific host use, but could not be justified with mtDNA divergence (Figures 1 &2). We are aware that simply challenging current taxonomic agreements does not improve this issue. With their remarkable reproductive mode variation and patterns of host specialization, these parasitoids clearly deserve further study. Treating them as a single unit under the umbrella '*Lysiphlebus fabarum *group' might be the least contentious approach for the time being.

The relationships of the outgroup taxa in our mitochondrial phylogeny were largely consistent with existing phylogenies of the Aphidiinae [111-113]. The only surprise was the placement of *Adialytus salicaphis *between Palearctic and Nearctic representatives of the genus *Lysiphlebus *according to both mitochondrial genes. This is in contrast to a previous phylogeny based on the mitochondrial 16S rRNA gene [111]. However, *A. salicaphis *used to be placed in the genus *Lysiphlebus *[e.g. [108]], and Sanchis *et al*. [114] also found a member of *Adialytus *falling inside the genus *Lysiphlebus *using nuclear 18S rRNA while others branched outside. It is thus recommended that the phylogenetic status of *Adialytus *be revisited.

## Conclusions

The *L. fabarum *group is an evolutionarily young sexual-asexual complex of aphid parasitoids with incomplete genetic isolation between reproductive modes. We inferred three mechanistic pathways which may give rise to new thelytokous lineages and/or mediate gene flow between thelytokous and arrhenotokous wasps: (*i*) introgression from sexuals into asexuals through matings between sexual males and thelytokous females, (*ii*) the formation of new asexual lineages via 'contagious parthenogenesis', and (*iii*) 'cryptic sex' within asexuals. The latter two routes rely on rare males that thelytokous lines are known to produce spontaneously. Probably all three mechanisms of genetic exchange operate jointly in generating the high genotypic diversity observed in asexual parasitoids, although their relative importance appears to differ among populations. In addition, there is clear evidence for host specialization in the *L. fabarum *group. It has resulted in partially strong differentiation among wasps collected from different aphids, which exceeds the differentiation between the three morpholocially defined species. This, the shallow topology of the mitochondrial tree and the finding that the characters used in taxonomy are phylogenetically non-conservative all indicate that the current division into three species cannot be upheld.

## Authors' contributions

Project conception: CS CV. Field sampling: CS CV. Laboratory work, data generation and processing: CS. Data analysis: CS BES CV. Figure design: CS. Wrote the paper: CS CV. All authors improved and approved the final manuscript.

## Supplementary Material

Additional file 1 **Distribution of Lysiphlebus fabarum group reproductive modes across Europe**. Figure S1: Map of European sample locations and distribution of reproductive modes of Lysiphlebus parasitoids. Click here for file

Additional file 2 **Geographic distribution of reproductive modes of the Lysiphlebus fabarum group associated with Aphis hederae hosts**. Figure S2: Bar plots depicting the numbers of arrhenotokous and thelytokous Lysiphlebus fabarum group parasitoid samples collected at individual locations. Click here for file

Additional file 3 **Outgroup taxa**. Table S1: Detailed sampling and sequence information on the outgroup taxa used in the phylogenetic analyses (Figure 1). Click here for file

Additional file 4 **GenBank accession numbers of COΙ and ATP6 sequences of the Lysiphlebus fabarum group**. Table S2: Combination of individual gene's haplotype sequences corresponding to the concatenated sequence haplotype data. Click here for file

Additional file 5 **Overall nuclear genetic differentiation of the Lysiphlebus fabarum group**. Table S3: Pairwise F_ST _comparison among members of the Lysiphlebus fabarum group. Click here for file

## References

[B1] Normark BB Judson OP Moran NA Genomic signatures of ancient asexual lineages Biological Journal of the Linnean Society 2003 79 1 69 84

[B2] Schurko AM Neiman M Logsdon JM Signs of sex: what we know and how we know it Trends in Ecology & Evolution 2009 24 4 208 217 10.1016/j.tree.2008.11.010 19282047

[B3] Judson OP Normark BB Ancient asexual scandals Trends in Ecology & Evolution 1996 11 2 A41 A46 10.1016/0169-5347(96)81040-8 21237759

[B4] West SA Lively CM Read AF A pluralist approach to sex and recombination Journal of Evolutionary Biology 1999 12 6 1003 1012

[B5] Simon JC Delmotte F Rispe C Crease T Phylogenetic relationships between parthenogens and their sexual relatives: the possible routes to parthenogenesis in animals Biological Journal of the Linnean Society 2003 79 1 151 163

[B6] Simon JC Rispe C Sunnucks P Ecology and evolution of sex in aphids Trends in Ecology & Evolution 2002 17 1 34 39

[B7] Cook JM Sex determination in the Hymenoptera-a review of models and evidence Heredity 1993 71 4 421 435

[B8] Belshaw R Quicke DLJ Völkl W Godfray HCJ Molecular markers indicate rare sex in a predominantly asexual parasitoid wasp Evolution 1999 53 4 1189 1199 10.1111/j.1558-5646.1999.tb04532.x 28565514

[B9] Starý P Biology and distribution of microbe-associated thelytokous populations of aphid parasitoids (Hymenoptera, Braconidae, Aphidiinae) Journal of Applied Entomology 1999 123 4 231 235

[B10] Suomalainen E Saura A Lokki J Cytology and Evolution in Parthenogenesis 1987 CRC Press Boca Raton, Fla

[B11] Stouthamer R Breeuwer JAJ Hurst GDD *Wolbachia pipientis*: microbial manipulator of arthropod reproduction Annu Rev Microbiol 1999 53 71 102 10.1146/annurev.micro.53.1.71 10547686

[B12] Giorgini M Monti MM Caprio E Stouthamer R Hunter MS Feminization and the collapse of haplodiploidy in an asexual parasitoid wasp harboring the bacterial symbiont *Cardinium* Heredity 2009 102 4 365 371 10.1038/hdy.2008.135 19190669

[B13] Hagimori T Abe Y Date S Miura K The first finding of a *Rickettsia *bacterium associated with parthenogenesis induction among insects Curr Microbiol 2006 52 2 97 101 10.1007/s00284-005-0092-0 16450063

[B14] West SA Cook JM Werren JH Godfray HCJ *Wolbachia *in two insect host-parasitoid communities Molecular Ecology 1998 7 11 1457 1465 10.1046/j.1365-294x.1998.00467.x 9819901

[B15] Belshaw R Quicke DLJ The cytogenetics of thelytoky in a predominantly asexual parasitoid wasp with covert sex Genome 2003 46 1 170 173 10.1139/g02-112 12669810

[B16] Beukeboom LW Pijnacker LP Automictic parthenogenesis in the parasitoid *Venturia canescens *(Hymenoptera: Ichneumonidae) revisited Genome 2000 43 6 939 944 10.1139/g00-061 11195346

[B17] Sandrock C Vorburger C Single-locus recessive inheritance of asexual reproduction in a parasitoid wasp Current Biology 2011 21 5 433 437 10.1016/j.cub.2011.01.070 21353557

[B18] Lattorff HMG Moritz RFA Fuchs S A single locus determines thelytokous parthenogenesis of laying honeybee workers (*Apis mellifera capensis*) Heredity 2005 94 5 533 537 10.1038/sj.hdy.6800654 15741997

[B19] Innes DJ Hebert PDN The origin and genetic basis of obligate parthenogenesis in *Daphnia pulex* Evolution 1988 42 5 1024 1035 10.1111/j.1558-5646.1988.tb02521.x 28581165

[B20] Paland S Colbourne JK Lynch M Evolutionary history of contagious asexuality in *Daphnia pulex* Evolution 2005 59 4 800 813 15926690

[B21] Stelzer CP Schmidt J Wiedlroither A S R Loss of sexual reproduction and dwarfing in a small metazoan PLoS One 2010 5 9 e12854 10.1371/journal.pone.0012854 PMC2942836 20862222

[B22] Starý P Aphid Parasitiods of the Czech Republik Academia, Prague, Czech Republik 2006

[B23] Kavallieratos NG Tomanović Ž Starý P Athanassiou CG Sarlis GP Petrović O Niketić M Veroniki MA A survey of aphid parasitoids (Hymenoptera: Braconidae: Aphidiinae) of Southeastern Europe and their aphid-plant associations Applied Entomology and Zoology 2004 39 3 527 563

[B24] Starý P Specificity of parasitoids (Hymenoptera, Aphidiidae) to the black bean aphid, *Aphis fabae *complex, in agroecosystems Acta Entomologica Bohemoslovaca 1986 83 1 24 29

[B25] Morehead SA Seger J Feener DH Brown BV Evidence for a cryptic species complex in the ant parasitoid *Apocephalus paraponerae *(Diptera: Phoridae) Evolutionary Ecology Research 2001 3 3 273 284

[B26] Smith MA Woodley NE Janzen DH Hallwachs W Hebert PDN DNA barcodes reveal cryptic host-specificity within the presumed polyphagous members of a genus of parasitoid flies (Diptera: Tachinidae) Proceedings of the National Academy of Sciences of the United States of America 2006 103 10 3657 3662 10.1073/pnas.0511318103 PMC1383497 16505365

[B27] Smith MA Rodriguez JJ Whitfield JB Deans AR Janzen DH Hallwachs W Hebert PDN Extreme diversity of tropical parasitoid wasps exposed by iterative integration of natural history, DNA barcoding, morphology, and collections Proceedings of the National Academy of Sciences of the United States of America 2008 105 34 12359 12364 10.1073/pnas.0805319105 PMC2518452 18716001

[B28] Kankare M Van Nouhuys S Hanski I Genetic divergence among host-specific cryptic species in *Cotesia melitaearum *aggregate (Hymenoptera: Braconidae), parasitoids of checkerspot butterflies Annals of the Entomological Society of America 2005 98 3 382 394

[B29] Stireman JO Nason JD Heard SB Seehawer JM Cascading host-associated genetic differentiation in parasitoids of phytophagous insects Proceedings of the Royal Society B-Biological Sciences 2006 273 1586 523 530 10.1098/rspb.2005.3363 PMC1560066 16537122

[B30] Kolaczan CR Heard SB Segraves KA Althoff DM Nason JD Spatial and genetic structure of host-associated differentiation in the parasitoid *Copidosoma gelechiae* Journal of Evolutionary Biology 2009 22 6 1275 1283 10.1111/j.1420-9101.2009.01742.x 19453371

[B31] Baer CF Tripp DW Bjorksten TA Antolin MF Phylogeography of a parasitoid wasp (*Diaeretiella rapae*): no evidence of host-associated lineages Molecular Ecology 2004 13 7 1859 1869 10.1111/j.1365-294X.2004.02196.x 15189209

[B32] Althoff DM A test of host-associated differentiation across the 'parasite continuum' in the tri-trophic interaction among yuccas, bogus yucca moths, and parasitoids Molecular Ecology 2008 17 17 3917 3927 10.1111/j.1365-294X.2008.03874.x 18662219

[B33] Lozier JD Roderick GK Mills NJ Molecular markers reveal strong geographic, but not host associated, genetic differentiation in *Aphidius transcaspicus*, a parasitoid of the aphid genus *Hyalopterus* Bull Entomol Res 2009 99 1 83 96 10.1017/S0007485308006147 18662432

[B34] Cronin JT Abrahamson AG Do parasitoids diversify in response to host-plant shifts by herbivorous insects? Ecological Entomology 2001 26 4 347 355

[B35] Völkl W Mackauer M Interactions between ants and parasitoid wasps foraging for *Aphis fabae *spp. *cirsiiacanthoidis *on thistles J Insect Behav 1993 6 3 301 312

[B36] Liepert C Chemische Mimikry bei Blattlausparasitoiden der Gattung *Lysiphlebus *(Hymenoptera, Aphidiidae) PhD Thesis 1996 Universität Bayreuth, Germany

[B37] R Development Core Team R: A language and environment for statistical computing 2008 http://cran.r-project.org

[B38] Crawley MJ Statistics: An Introduction using R 2005 Cichester: John Wiley & Sons Ltd

[B39] Sandrock C Frauenfelder N von Burg S Vorburger C Microsatellite DNA markers for the aphid parasitoid *Lysiphlebus fabarum *and their applicability to related species Mol Ecol Notes 2007 7 6 1080 1083

[B40] Fauvergue X Tentelier C Genson G Audiot P Guillemaud T Streiff RJ Microsatellite DNA markers for *Lysiphlebus testaceipes* Mol Ecol Notes 2005 5 1 109 111

[B41] Arnaud-Haond S Belkhir K GENCLONE: a computer program to analyse genotypic data, test for clonality and describe spatial clonal organization Mol Ecol Notes 2007 7 1 15 17

[B42] Arnaud-Haond S Duarte CM Alberto F Serrao EA Standardizing methods to address clonality in population studies Molecular Ecology 2007 16 24 5115 5139 10.1111/j.1365-294X.2007.03535.x 17944846

[B43] Dorken ME Eckert CG Severely reduced sexual reproduction in northern populations of a clonal plant, *Decodon verticillatus *(Lythraceae) Journal of Ecology 2001 89 3 339 350

[B44] Excoffier L Laval G Schneider S Arlequin (version 3.0): An integrated software package for population genetics data analysis Evol Bioinform 2005 47 50 PMC2658868 19325852

[B45] Goudet J FSTAT (version 2.9.3.2), a program to estimate and test gene diversities and fixation indices 2001 http://www2.unil.ch/popgen/softwares/fstat.htm

[B46] Engelstädter J Constraints on the evolution of asexual reproduction BioEssays 2008 30 11-12 1138 1150 10.1002/bies.20833 18937362

[B47] Sunnucks P De Barro PJ Lushai G Maclean N Hales DF Genetic structure of an aphid studied using microsatellites: cyclic parthenogenesis, differentiated lineages and host specialization Molecular Ecology 1997 6 11 1059 1073 10.1046/j.1365-294x.1997.00280.x 9394464

[B48] Halkett F Simon JC Balloux F Tackling the population genetics of clonal and partially clonal organisms Trends in Ecology & Evolution 2005 20 4 194 201 10.1016/j.tree.2005.01.001 16701368

[B49] Rice WR Analyzing tables of statistical tests Evolution 1989 43 223 225 10.1111/j.1558-5646.1989.tb04220.x 28568501

[B50] Folmer O Black M Hoeh W Lutz R Vrijenhoek RC DNA primers for amplification of mitochondrial cytochrome *c *oxidase subunit 1 from diverse metazoan invertebrates Mol Mar Biol Biotechnol 1994 3 294 299 7881515

[B51] Hebert PDN Cywinska A Ball SL deWaard JR Biological identifications through DNA barcodes Proc R Soc Lond Ser B-Biol Sci 2003 270 1512 313 321 10.1098/rspb.2002.2218 PMC1691236 12614582

[B52] Thompson JD Higgins DG Gibson TJ Clustal-W-Improving the sensitivity of progressive multiple sequence alignment through sequence weighting, position-specific gap penalties and weight matrix choice Nucleic Acids Research 1994 22 22 4673 4680 10.1093/nar/22.22.4673 PMC308517 7984417

[B53] Hall TA BioEdit: a user-friendly biological sequence alignment editor and analysis program for Windows 95/98/NT Nuc Acids Symp Ser 1999 41 95 98

[B54] Tamura K Dudley J Nei M Kumar S MEGA4: Molecular evolutionary genetics analysis (MEGA) software version 4.0 Molecular Biology and Evolution 2007 24 8 1596 1599 10.1093/molbev/msm092 17488738

[B55] Zhang DX Hewitt GM Nuclear integrations-challenges for mitochondrial-DNA markers Trends in Ecology and Evolution 1996 11 6 247 251 10.1016/0169-5347(96)10031-8 21237827

[B56] Song H Buhay JE Whiting MF Crandall KA Many species in one: DNA barcoding overestimates the number of species when nuclear mitochondrial pseudogenes are coamplified Proceedings of the National Academy of Sciences of the United States of America 2008 105 36 13486 13491 10.1073/pnas.0803076105 PMC2527351 18757756

[B57] Librado P Rozas J DnaSP v5: a software for comprehensive analysis of DNA polymorphism data Bioinformatics 2009 25 11 1451 1452 10.1093/bioinformatics/btp187 19346325

[B58] Swofford DL PAUP*. Phylogenetic Analysis using Parsimony (*and other Methods). Version 4 Sunderland, Massachusetts: Sinauer Associates 2003

[B59] Zwickl DJ Genetic algorithm approaches for the phylogenetic analysis of large biological sequence datasets under the maximum likelihood criterion PhD Thesis 2006 Austin, TX, USA: The University of Texas, Austin, TX, USA

[B60] Ronquist F Huelsenbeck JP MrBayes 3: Bayesian phylogenetic inference under mixed models Bioinformatics 2003 19 12 1572 1574 10.1093/bioinformatics/btg180 12912839

[B61] Lanave C Preparata G Saccone C Serio G A new method for calculating evolutionary substitution rates Journal of Molecular Evolution 1984 20 1 86 93 10.1007/BF02101990 6429346

[B62] Rodriguez F Oliver JL Marin A Medina JR The general stochastic-model of nucleotide substitution J Theor Biol 1990 142 4 485 501 10.1016/s0022-5193(05)80104-3 2338834

[B63] Posada D Crandall KA MODELTEST: testing the model of DNA substitution Bioinformatics 1998 14 9 817 818 10.1093/bioinformatics/14.9.817 9918953

[B64] Wilgenbusch J Warren D Swofford DL Awty: A system for graphical exploration of mcmc convergence in bayesian phylogenetic inference 2004 http://ceb.csit.fsu.edu/awty/10.1093/bioinformatics/btm388 17766271

[B65] Rambaut A Drummond AJ Tracer v1.4 2007 http://tree.bio.ed.ac.uk/software/tracer/

[B66] Maddison WP Maddison DR Mesquite: a modular system for evolutionary analysis. v2.71 2009 http://mesquiteproject.org

[B67] Lewis PO A likelihood approach to estimating phylogeny from discrete morphological character data Syst Biol 2001 50 6 913 925 10.1080/106351501753462876 12116640

[B68] Clement M Posada D Crandall KA TCS: a computer program to estimate gene genealogies Molecular Ecology 2000 9 10 1657 1659 10.1046/j.1365-294x.2000.01020.x 11050560

[B69] Lynch M Crease TJ The analysis of population survey data on DNA-sequence variation Molecular Biology and Evolution 1990 7 4 377 394 10.1093/oxfordjournals.molbev.a040607 1974693

[B70] Hudson RR Boos DD Kaplan NL A statistical test for detecting geographic subdivision Molecular Biology and Evolution 1992 9 1 138 151 10.1093/oxfordjournals.molbev.a040703 1552836

[B71] de Meeûs T Lehmann L Balloux F Molecular epidemiology of clonal diploids: a quick overview and a short DIY (do it yourself) notice Infection Genetics and Evolution 2006 6 2 163 170 10.1016/j.meegid.2005.02.004 16290062

[B72] de Meeûs T McCoy KD Prugnolle F Chevillon C Durand P Hurtrez-Bousses S Renaud F Population genetics and molecular epidemiology or how to "débusquer la bête" Infection Genetics and Evolution 2007 7 2 308 332 10.1016/j.meegid.2006.07.003 16949350

[B73] Belkhir K Borsa P Chikhi L Raufaste N Bonhomme F GENETIX 4.05, logiciel sous Windows TM pour la génétique des populations (Laboratoire Génome, Populations, Interactions, CNRS UMR 5000, Université de Montpellier II, Montpellier (France)) 1996

[B74] Smouse PE Long JC Sokal RR Multiple-regression and correlation extensions of the Mantel test of matrix correspondence Systematic Zoology 1986 35 4 627 632

[B75] Rousset F Genetic differentiation and estimation of gene flow from *F*-statistics under isolation by distance Genetics 1997 145 4 1219 1228 10.1093/genetics/145.4.1219 PMC1207888 9093870

[B76] Chakraborty R Jin L Pena SDJ, Chakraborty R, Epplen JT, Jeffreys AJ A unified approach to the study of hypervariable polymorphisms: statistical considerations of determining relatedness and population distances DNA Fingerprinting: State of the Science 1993 Basel, Switzerland: Birkhäuser 153 175 10.1007/978-3-0348-8583-6_14 8400687

[B77] Langella O Populations 1.2.30 1999 http://bioinformatics.org/~tryphon/populations/.

[B78] Nemec V Starý P Population diversity in deuterotokous *Lysiphlebus *species, parasitoids of aphids (Hymenoptera, Aphidiidae) Acta Entomologica Bohemoslovaca 1985 82 3 170 174

[B79] Nyabuga FN Loxdale HD Heckel DG Weisser WW Spatial population dynamics of a specialist aphid parasitoid, *Lysiphlebus hirticornis *Mackauer (Hymenoptera: Braconidae: Aphidiinae): evidence for philopatry and restricted dispersal Heredity 2010 105 5 433 442 10.1038/hdy.2009.190 20104237

[B80] Schneider MV Beukeboom LW Driessen G Lapchin L Bernstein C van Alphen JJM Geographical distribution and genetic relatedness of sympatrical thelytokous and arrhenotokous populations of the parasitoid *Venturia canescens *(Hymenoptera) Journal of Evolutionary Biology 2002 15 2 191 200

[B81] Schneider MV Driessen G Beukeboom LW Boll R van Eunen K Selzner A Talsma J Lapchin L Gene flow between arrhenotokous and thelytokous populations of *Venturia canescens *(Hymenoptera) Heredity 2003 90 3 260 267 10.1038/sj.hdy.6800245 12634810

[B82] Rey O Loiseau A Facon B Foucaud J Orivel J Cornuet JM Robert S Dobigny G Delabie JHC Dos Santos C Meiotic recombination dramatically decreased in thelytokous queens of the little fire ant and their sexually produced workers Molecular Biology and Evolution 2011 28 9 2591 2601 10.1093/molbev/msr082 21459760

[B83] de Boer JG Ode PJ Vet LEM Whitfield J Heimpel GE Diploid males sire triploid daughters and sons in the parasitoid wasp *Cotesia vestalis* Heredity 2007 99 3 288 294 10.1038/sj.hdy.6800995 17551527

[B84] Heimpel GE de Boer JG Sex determination in the Hymenoptera Annu Rev Entomol 2008 53 209 230 10.1146/annurev.ento.53.103106.093441 17803453

[B85] Engelstädter J Sandrock C Vorburger C Contagious parthenogenesis, automixis, and a sex determination meltdown Evolution 2011 65 2 501 511 10.1111/j.1558-5646.2010.01145.x 21029077

[B86] Storeck A Poppy GM van Emden HF Powell W The role of plant chemical cues in determining host preference in the generalist aphid parasitoid *Aphidius colemani* Entomologia Experimentalis Et Applicata 2000 97 1 41 46

[B87] Daza-Bustamante P Fuentes-Contreras E Rodriguez LC Figueroa CC Niemeyer HM Behavioural differences between *Aphidius ervi *populations from two tritrophic systems are due to phenotypic plasticity Entomologia Experimentalis Et Applicata 2002 104 2-3 321 328

[B88] Henry LM Roitberg BD Gillespie DR Host-range evolution in *Aphidius *parasitoids: fidelity, virulence and fitness trade-offs on an ancestral host Evolution 2008 62 3 689 699 10.1111/j.1558-5646.2007.00316.x 18182071

[B89] Vet LEM Dicke M Ecology of infochemical use by natural enemies in a tritrophic context Annu Rev Entomol 1992 37 141 172

[B90] Steinberg S Prag H Rosen D Host plant fitness and host acceptance in the aphid parasitoid *Lysiphlebus testaceipes *(Cresson) Bulletin IOBC wprs 1993 16 161 164

[B91] Weisser WW Völkl W Dispersal in the aphid parasitoid, *Lysiphlebus cardui *(Marshall) (Hymenoptera: Aphidiidae) Journal of Applied Entomology 1997 121 1 23 28

[B92] Antolin MF Bjorksten TA Vaughn TT Host-related fitness trade-offs in a presumed generalist parasitoid, *Diaeretiella rapae *(Hymenoptera: Aphidiidae) Ecological Entomology 2006 31 3 242 254

[B93] Carver M The potential host ranges in Australia of some imported aphid parasites [Hymenoptera, Ichneumonoidea, Aphidiidae] Entomophaga 1984 29 4 351 359

[B94] Mackauer M Wirtsbindung der Aphidiinae und Fahrholz'sche Regel Verh Int Kongr Entomol Wien, 1960 1962 2

[B95] Vandel A La parthénogenèse géographique. Contribution à l'étude biologique et cytologique de la parthénogenèse naturelle Bulletin Biologique de la France et de la Belgique 1928 62 164 281

[B96] Peck JR Yearsley JM Waxman D Explaining the geographic distributions of sexual and asexual populations Nature 1998 391 889 892

[B97] Stone G Atkinson R Rokas A Csoka G Nieves-Aldrey JL Differential success in northwards range expansion between ecotypes of the marble gallwasp *Andricus kollari*: a tale of two lifecycles Molecular Ecology 2001 10 3 761 778 10.1046/j.1365-294x.2001.01211.x 11298986

[B98] Maynard Smith J The Evolution of Sex 1978 Cambridge: Cambridge University Press

[B99] De Meester L Gomez A Okamura B Schwenk K The Monopolization Hypothesis and the dispersal-gene flow paradox in aquatic organisms Acta Oecol-Int J Ecol 2002 23 3 121 135

[B100] Barrett LG Thrall PH Burdon JJ Nicotra AB Linde CC Population structure and diversity in sexual and asexual populations of the pathogenic fungus *Melampsora lini* Molecular Ecology 2008 17 14 3401 3415 10.1111/j.1365-294X.2008.03843.x PMC2653454 18573166

[B101] Aslan MM Uygun N Starý P A survey of aphid parasitoids in Kahramanmaras, Turkey (Hymenoptera: Braconidae, Aphidiinae; and Hymenoptera: Aphelinidae) Phytoparasitica 2004 32 3 255 263

[B102] Rakhshani E Talebi AA Kavallieratos NG Rezwani A Manzari S Tomanović Ž Parasitoid complex (Hymenoptera, Braconidae, Aphidiinae) of *Aphis craccivora *Koch (Hemiptera: Aphidoidea) in Iran Journal of Pest Science 2005 78 4 193 198

[B103] Rakhshani E Talebi AA Starý P Tomanović Ž Manzari S Aphid-parasitoid (Hymenoptera, Braconidae, Aphidiinae) associations on willows and poplars in Iran Acta Zool Acad Sci Hung 2007 53 3 281 292

[B104] Tomanović Ž Kavallieratos NG Starý P Petrović-Obradović O Tomanović S Jovanović S Aphids and parasitoids on willows and poplars in southeastern Europe (Hornoptera: Aphidoidea; Hymenoptera: Braconidae, Aphidiinae) J Plant Dis Prot 2006 113 4 174 180

[B105] Rokas A Atkinson RJ Webster L Csoka G Stone GN Out of Anatolia: longitudinal gradients in genetic diversity support an eastern origin for a circum-Mediterranean oak gallwasp *Andricus quercustozae* Molecular Ecology 2003 12 8 2153 2174 10.1046/j.1365-294x.2003.01894.x 12859636

[B106] Mackauer M Die europäischen Arten der Gattung *Lysiphlebus *Förster (Hymenoptera: Braconidae, Aphidiinae). Eine monographische Revision Beiträge zur Entomologie 1960 10 582 623

[B107] Starý P Two new *Lysiphlebus *species from Europe Acta Entomologica Bohemoslovaca 1985 82 6 426 430

[B108] Starý P Aphid parasites (Hymenoptera: Aphidiidae) of the Mediterranean area 1976 Junk, The Hague, The Netherlands

[B109] Tremblay E Eady RD *Lysiphlebus confusus *n.sp. per *Lysiphlebus ambiguus *sensu Auct Boll Lab Entomol Agrar Filippo Silvestri 1978 35 180 184

[B110] Hebert PDN Ratnasingham S deWaard JR Barcoding animal life: cytochrome c oxidase subunit 1 divergences among closely related species Proc R Soc Lond Ser B-Biol Sci 2003 270 S96 S99 10.1098/rsbl.2003.0025 PMC1698023 12952648

[B111] Kambhampati S Völkl W Mackauer M Phylogenetic relationships among genera of Aphidiinae (Hymenoptera: Braconidae) based on DNA sequence of the mitochondrial 16S rRNA gene Systematic Entomology 2000 25 4 437 445

[B112] Smith PT Kambhampati S Völkl W Mackauer M A phylogeny of aphid parasitoids (Hymenoptera: Braconidae: Aphidiinae) inferred from mitochondrial NADH 1 dehydrogenase gene sequence Mol Phylogenet Evol 1999 11 2 236 245 10.1006/mpev.1998.0575 10191068

[B113] Belshaw R Quicke DLJ A molecular phylogeny of the Aphidiinae (Hymenoptera: Braconidae) Mol Phylogenet Evol 1997 7 3 281 293 10.1006/mpev.1996.0400 9187088

[B114] Sanchis A Latorre A Gonzalez-Candelas F Michelena JM An 18S rDNA-based molecular phylogeny of Aphidiinae (Hymenoptera: Braconidae) Mol Phylogenet Evol 2000 14 2 180 194 10.1006/mpev.1999.0701 10679154

