# Supplementary material for: Evolution of reproductive mode variation and host associations in a sexual-asexual complex of aphid parasitoids
Source: BMC Evol Biol. 2011 Dec 1;11:348. doi: 10.1186/1471-2148-11-348 (PMC3259107; doi:10.1186/1471-2148-11-348)

Additional file 1: Distribution of *Lysiphlebus fabarum* group reproductive modes across Europe.

Figure S1: Map of European sample locations and distribution of reproductive modes of *Lysiphlebus* parasitoids. Pie charts represent proportional distribution of arrhenotokous (black) and thelytokous (red) parasitoids of the *L. fabarum* group of corresponding locations (see Table 2).

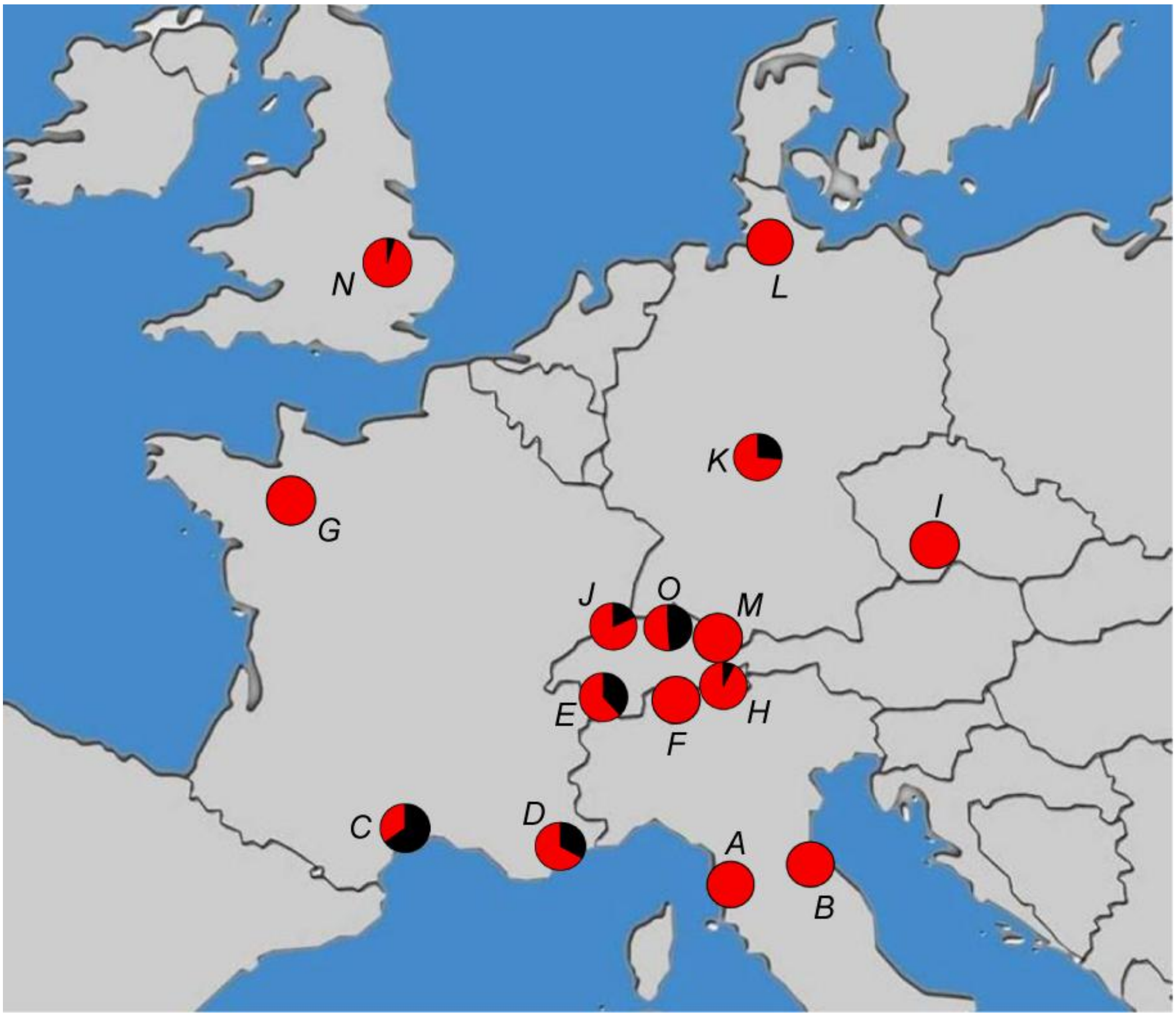

Supplement: Additional file 1 — Distribution of Lysiphlebus fabarum group reproductive modes across Europe. Figure S1: Map of European sample locations and distribution of reproductive modes of Lysiphlebus parasitoids. [file 1471-2148-11-348-S1.PDF]
