# Supplementary material for: Evolution of reproductive mode variation and host associations in a sexual-asexual complex of aphid parasitoids
Source: BMC Evol Biol. 2011 Dec 1;11:348. doi: 10.1186/1471-2148-11-348 (PMC3259107; doi:10.1186/1471-2148-11-348)

**Additional file 2: Geographic distribution of reproductive modes of the *Lysiphlebus fabarum* group associated with *Aphis hederae* hosts.**

**Figure S2: Arrhenotokous and thelytokous *Lysiphlebus fabarum* group parasitoids collected from *Aphis hederae* at individual locations.** Bar plots indicate total numbers of wasp samples from corresponding regions. Locations are arranged according to a south-north gradient. For details about sampling locations see Table 2 and Figure S1 [Additional file 1].

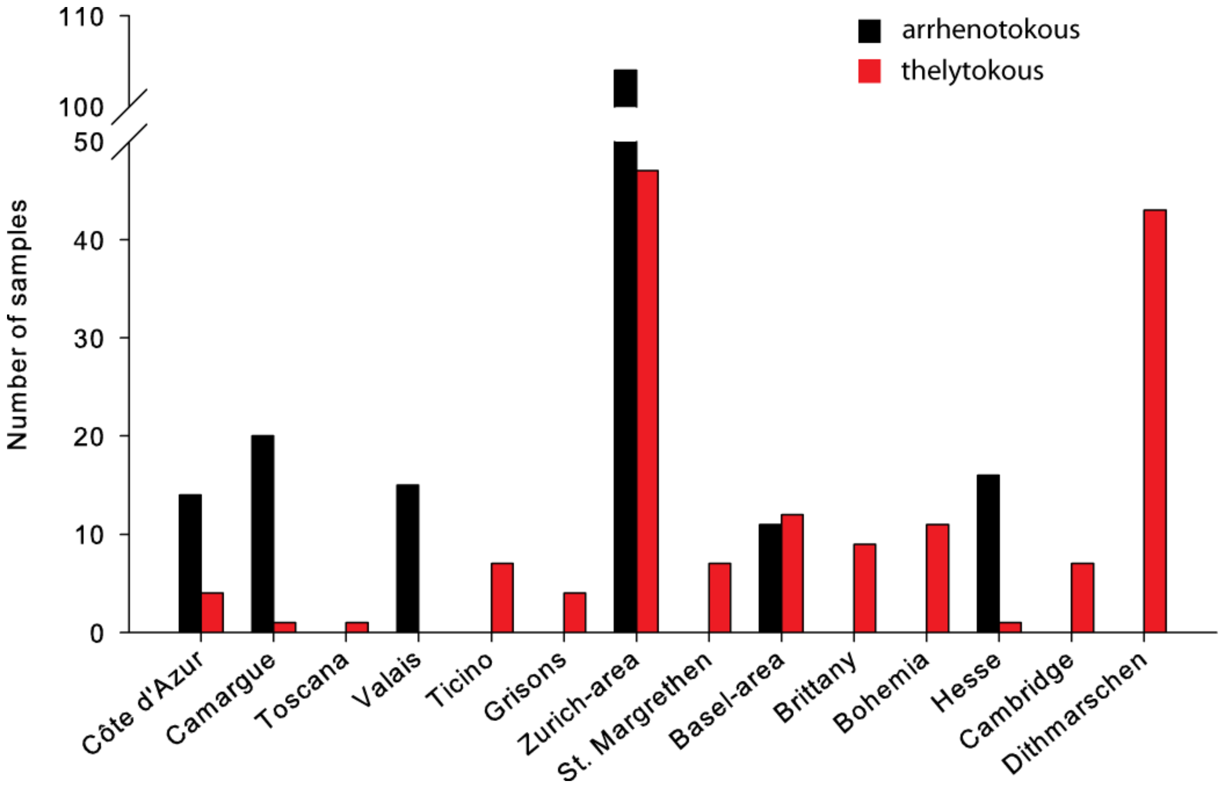

Supplement: Additional file 2 — Geographic distribution of reproductive modes of the Lysiphlebus fabarum group associated with Aphis hederae hosts. Figure S2: Bar plots depicting the numbers of arrhenotokous and thelytokous Lysiphlebus fabarum group parasitoid samples collected at individual locations. [file 1471-2148-11-348-S2.PDF]
