# Supplementary material for: Evolution of reproductive mode variation and host associations in a sexual-asexual complex of aphid parasitoids
Source: BMC Evol Biol. 2011 Dec 1;11:348. doi: 10.1186/1471-2148-11-348 (PMC3259107; doi:10.1186/1471-2148-11-348)
Supplement: Additional file 3 — Outgroup taxa. Table S1: Detailed sampling and sequence information on the outgroup taxa used in the phylogenetic analyses (Figure 1). [file 1471-2148-11-348-S3.PDF]

Additional file 3: Outgroup taxa.

Table S1: Detailed sampling and sequence information on the outgroup taxa used in the phylogenetic analyses (Figure 1). Geographic origins see Table 2. Both specimens of *L. hirticornis* from central and northern Germany shared the same haplotype. Three and two samples of *L. testaceipes* each shared identical haplotypes, both occurring at distantly separated regions in southern France, respectively.

			GenBank accession number	
Species	Host species	Geographic origin	COI	ATP6
Lysiphlebus hirticornis	*Metopeurum fuscoviride*	Hesse (D)	HQ724540	HQ724577
Lysiphlebus hirticornis	*Metopeurum fuscoviride*	Dithmarschen (D)	HQ724540	HQ724577
Lysiphlebus testaceipes[1]	*Aphis hederae*	Camargue (F)	HQ724537	HQ724574
Lysiphlebus testaceipes[1]	*Aphis ruborum*	Camargue (F)	HQ724537	HQ724574
Lysiphlebus testaceipes[1]	*Aphis fabae fabae*	Côte d'Azur (F)	HQ724537	HQ724574
Lysiphlebus testaceipes[2]	*Brachycaudus cardui*	Camargue (F)	HQ724538	HQ724575
Lysiphlebus testaceipes[2]	*Aphis sp.*	Côte d'Azur (F)	HQ724538	HQ724575
Adialytus salicaphis	*Chaitophorus sp.*	Zurich-area (CH)	HQ724539	HQ724576
Diaeretiella rapae	*Brevycoryne brassicae*	Zurich-area (CH)	HQ724536	HQ724573
Aphidius colemani	*Myzus persicae*	Zurich-area (CH)	HQ724535	HQ724572
