# Supplementary material for: Evolution of reproductive mode variation and host associations in a sexual-asexual complex of aphid parasitoids
Source: BMC Evol Biol. 2011 Dec 1;11:348. doi: 10.1186/1471-2148-11-348 (PMC3259107; doi:10.1186/1471-2148-11-348)
Supplement: Additional file 4 — GenBank accession numbers of COΙ and ATP6 sequences of the Lysiphlebus fabarum group. Table S2: Combination of individual gene's haplotype sequences corresponding to the concatenated sequence haplotype data. [file 1471-2148-11-348-S4.PDF]

Additional file 4: GenBank accession numbers of COI and ATP6 sequences of the *Lysiphlebus fabarum* group.

Within the *Lysiphlebus fabarum* group 22 distinct haplotypes for the COI gene were detected (GenBank accession numbers: HQ724541 – HQ724571), while 20 distinct sequences were found for the ATP6 gene (GenBank accession numbers: HQ724578 – HQ724606).

Table S2: Combination of individual gene's haplotype sequences corresponding to the concatenated sequence haplotype data. The 33 distinct total length sequence haplotypes (concatenated genes) of the *Lysiphlebus fabarum* group (see Figure 1) were composed as detailed. Note that in some instances multiple sequences for identical haplotypes have been deposited, each one for a different corresponding morphotype, i.e. traditionally recognized taxa.

Concatenated Sequence Haplotype	GenBank accession number	
	COI haplotype	ATP6 haplotype
1	HQ724549	HQ724581
2	HQ724549	HQ724578
3	HQ724550	HQ724578
4	HQ724546	HQ724578
5	HQ724549	HQ724580
6	HQ724548	HQ724578
7	HQ724541, HQ724542	HQ724584
8	HQ724541, HQ724542	HQ724586, HQ724587
9	HQ724541, HQ724542	HQ724585
10	HQ724543, HQ724544	HQ724586, HQ724587
11	HQ724541, HQ724542	HQ724582
12	HQ724547	HQ724583
13	HQ724557	HQ724579
14	HQ724541, HQ724542	HQ724579
15	HQ724565, HQ724566, HQ724567	HQ724599, HQ724600
16	HQ724560	HQ724599, HQ724600
17	HQ724559	HQ724599, HQ724600
18	HQ724565, HQ724566, HQ724567	HQ724595
19	HQ724565, HQ724566, HQ724567	HQ724596
20	HQ724563	HQ724601, HQ724602, HQ724603
21	HQ724565, HQ724566, HQ724567	HQ724601, HQ724602, HQ724603
22	HQ724562	HQ724601, HQ724602, HQ724603
23	HQ724561	HQ724601, HQ724602, HQ724603
24	HQ724565, HQ724566, HQ724567	HQ724597, HQ724598
25	HQ724564	HQ724601, HQ724602, HQ724603
26	HQ724558	HQ724592
27	HQ724569, HQ724570, HQ724571	HQ724588, HQ724589
28	HQ724569, HQ724570, HQ724571	HQ724590, HQ724591
29	HQ724568	HQ724594
30	HQ724545	HQ724594
31	HQ724555	HQ724594
32	HQ724552, HQ724553, HQ724554	HQ724604, HQ724605, HQ724606
33	HQ724556	HQ724593
