# Supplementary material for: Evolution of reproductive mode variation and host associations in a sexual-asexual complex of aphid parasitoids
Source: BMC Evol Biol. 2011 Dec 1;11:348. doi: 10.1186/1471-2148-11-348 (PMC3259107; doi:10.1186/1471-2148-11-348)
Supplement: Additional file 5 — Overall nuclear genetic differentiation of the Lysiphlebus fabarum group. Table S3: Pairwise FST comparison among members of the Lysiphlebus fabarum group. [file 1471-2148-11-348-S5.PDF]

Additional file 5: Overall nuclear genetic differentiation of the *Lysiphlebus fabarum* group.

Table S3: **Pairwise F_{ST} comparison among members of the *Lysiphlebus fabarum* group.** Host affiliated groups are categorized within morphotypes corresponding to both reproductive modes (definitions see Tables 1 & 2). Overall numbers of locations (Area) and microsatellite multi locus genotypes (MLG) are indicated. Pairwise F_{ST} among groups (above diagonal) and among locations within groups (diagonal, in italics) are shown with significant differentiation highlighted in bold.

Mode				Sex				Asex															
	Morph			*Lfa*			*Lco*	*Lfa*					*Lco*						*Lca*				
		Host		*Ahe*	*Aru*	*Bca*	*Afc*	*Aff*	*Afc*	*Aur*	*Ahe*	*Aru*	*Aff*	*Afc*	*Aur*	*Ahe*	*Aru*	*Afa*	*Aff*	*Afc*	*Aur*	*Ahe*	*Aru*
			Area	6	2	4	1	14	9	8	11	12	4	4	4	7	13	8	7	9	7	3	3
			MLG	180	5	14	3	46	25	16	46	36	8	5	5	9	35	21	20	24	10	3	4
Sex	*Lfa*	*Ahe*		***.208***	**.108**	**.317**	**.357**	**.249**	**.285**	**.294**	**.118**	**.299**	**.267**	**.250**	**.243**	**.287**	**.343**	**.341**	**.256**	**.248**	**.250**	**.231**	**.240**
		Aru			***.444***	**.524**	**.349**	**.145**	**.200**	**.236**	**.054**	**.207**	**.186**	**.161**	**.188**	**.209**	**.271**	**.331**	**.174**	**.169**	**.166**	**.136**	**.151**
		Bca				***.108***	**.649**	**.385**	**.457**	**.409**	**.321**	**.385**	**.449**	**.439**	**.454**	**.412**	**.394**	**.495**	**.405**	**.405**	**.478**	**.521**	**.456**
	Lco	*Afc*					-	**.214**	**.260**	**.309**	**.203**	**.212**	**.265**	**.301**	.311	**.334**	**.336**	**.384**	**.255**	**.250**	**.267**	.266	.258
Asex	*Lfa*	*Aff*						***.076***	-.009	.010	**.048**	.087	**.173**	.098	.067	**.143**	**.158**	**.140**	**.101**	**.083**	**.102**	.011	.071
		Afc							***.136***	.016	**.068**	**.118**	**.233**	**.146**	**.106**	**.191**	**.191**	**.148**	**.120**	**.100**	**.136**	.040	**.113**
		Aur								*-.065*	**.078**	**.134**	**.273**	**.207**	**.071**	**.237**	**.212**	**.212**	**.190**	**.163**	**.176**	**.136**	**.146**
		Ahe									***.111***	**.117**	**.142**	**.065**	**.038**	**.132**	**.170**	**.160**	**.085**	**.080**	**.077**	.005	**.024**
		Aru										***.119***	**.103**	**.063**	.076	**.066**	**.063**	**.188**	**.165**	**.143**	**.159**	**.092**	**.082**
	Lco	*Aff*											*.110*	.029	**.139**	.023	**.157**	**.307**	**.185**	**.199**	**.199**	.150	.124
		Afc												*-.327*	.011	-.027	.053	**.202**	**.069**	**.091**	**.113**	-.002	-.023
		Aur													*-.246*	.058	.060	**.171**	**.137**	**.135**	**.122**	.052	-.033
		Ahe														***.308***	.023	**.222**	**.165**	**.171**	**.204**	**.124**	.081
		Aru															*.072*	**.209**	**.203**	**.197**	**.220**	**.157**	.070
		Afa																***.116***	**.225**	**.184**	**.254**	**.126**	**.161**
	Lca	*Aff*																	***.115***	.014	.014	-.079	.010
		Afc																		***.272***	.009	-.110	.020
		Aur																			*-.217*	-.076	-.007
		Ahe																				-	-.096
		Aru																					*-.122*
